# Pd/C-catalyzed aerobic oxidative esteriﬁcation of alcohols and aldehydes: a highly efficient microwave-assisted green protocol

**DOI:** 10.3762/bjoc.10.149

**Published:** 2014-06-26

**Authors:** Marina Caporaso, Giancarlo Cravotto, Spyros Georgakopoulos, George Heropoulos, Katia Martina, Silvia Tagliapietra

**Affiliations:** 1 Dipartimento di Scienza e Tecnologia del Farmaco, University of Turin, Via P. Giuria 9, 10125 Torino, Italy. Fax +390116707687; Tel: +390116707684; 2 Interdepartmental Centre “Nanostructured interfaces and surfaces NIS, University of Turin, Via P. Giuria 7–9, 10125 Torino, Italy; 3 Institute of Biology, Medicinal Chemistry and Biotechnology, National Hellenic Research Foundation, 48. Vas. Constantinou Ave., 11635 Athens, Greece

**Keywords:** aerobic oxidation, alcohol, esterification, heterogeneous catalysis, microwaves

## Abstract

We herein describe an environmentally friendly microwave-assisted oxidative esteriﬁcation of alcohols and aldehydes in the presence of molecular oxygen and a heterogeneous catalysis (Pd/C, 5 mol %). This efficient and ligandless conversion procedure does not require the addition of an organic hydrogen acceptor. The reaction rate is strongly enhanced by mild dielectric heating. Furthermore, it is a versatile green procedure which generally enables the isolation of esters to be carried out by simple filtration in almost quantitative yields.

## Introduction

Selective oxidations of alcohols are some of the most important transformations in organic synthesis. Therefore, reactions that employ reusable heterogeneous catalysts and molecular oxygen are highly desirable from atom economy and environmental impact point of view [1–3].

A number of methods have been developed for the aerobic oxidation of primary and secondary alcohols to aldehydes and ketones using Au [4–8], Pd [9–12], Ru [13–16] and Cu [17–19] supported catalysts.

**Scheme 1:**
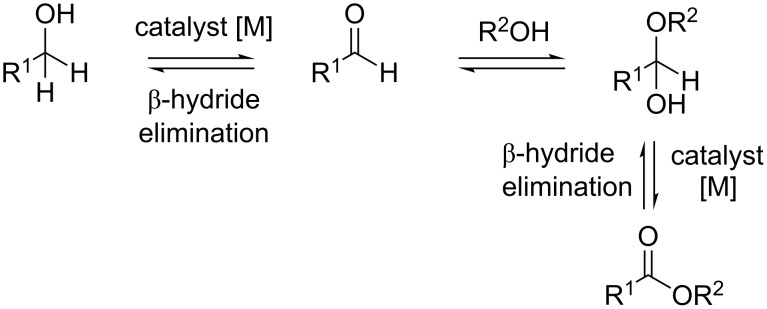
Reaction pathway of aerobic oxidative esteriﬁcation of alcohols.

An elegant approach to the direct oxidative esteriﬁcation of alcohols has been described in recent years [20]. The proposed reaction pathway (Scheme 1) is based on the oxidation of the alcohol to an aldehyde, followed by conversion to the respective hemiacetal, which is further oxidized to the corresponding ester. Relatively little work has been carried out on this so far, besides some remarkable results on the catalytic performance of Au [21–26], Ru [27–29], Ir [30] and Pd [31–35]. Moreover, the search for new, less expensive and environmentally friendly catalysts has attracted a great deal of interest because of the limited availability and high price of noble metals.

Homogeneous and heterogeneous Pd catalysts have been widely used in the selective oxidation of alcohols [36–37]. Most publications have focused on the conversion of alcohol to aldehydes or ketones [38–39]. Several examples of the heterogeneous oxidative esterification of alcohols in the presence of molecular oxygen or air have been described [23,25,40–43], however, the main limit of this process is its lack of selectivity, as has already been reported. Several Pd-catalyzed procedures have been optimized and Ag [31–32] or Bi salts [40], hydrosilanes [33] or specially designed electron-deficient ligands [9,31] have all been added to the catalyst in an attempt to improve conversion and selectivity. Other examples refer to the use of Pd(II) species in the presence of a cooxidant such as benzyl chloride or desyl chloride [34,44]. The optimized procedures are therefore limited with respect to catalyst accessibility and long reaction time. For this reason, the use of inexpensive and easily reusable catalysts as well as a reduction in its long reaction time can make this process cost-effective and environmentally benign.

In comparison with more expensive and air-sensitive Pd catalysts, the common Pd/C is easily handled and filtered off from the reaction mixture. It is remarkably stable under acidic and basic conditions and features a much higher surface area than alumina- and silica-supported catalysts [45]. Moreover, charcoal is rapidly heated under microwave (MW) irradiation thanks to its high electrical and thermal conductivity [46–47]. It is now widely accepted that MW can dramatically enhance reaction rates in organic synthesis [48], while the kinetics and yields of any chemical modification are strongly improved by the optimal heat transfer provided by selective volumetric dielectric heating. MW is surely one of the best techniques for cutting down reaction time, minimizing catalyst loading and reducing reagent excess [49–50]. Design improvements in commercial MW ovens for organic synthesis have recently brought to the market high power (up to 1.5 kW) pressure resistant reactors (up to 200 bar) that are equipped with separate multiple gas inlets and can be considered autoclaves. Only a few publications have described this technique in heterogeneous gas-phase reactions that are important for industrial processes [51–55]. We have recently displayed the versatility of these reactors in promoting reactions with gaseous reagents in a closed cavity [56]. In this present work, we report an efficient Pd/C-catalyzed aerobic oxidative esterification of aldehydes and alcohols in the presence of molecular oxygen in a closed MW reactor. The optimized procedure does not require co-catalysts, co-oxidants or ligands.

## Results and Discussion

Pd-catalyzed esterification can be performed with Pd(II) species [31–35,44]. The challenge of performing aerobic oxidation under MW irradiation in a preliminary investigation prompted us to select benzylalcohol and methanol as substrate for the model reaction and PdCl_2_(PPh_3_)_2_ and Pd(OAc)_2_ as the catalysts. The reactions were performed in a professional multimode reactor (SynthWave-Milestone/MLS) which was equipped with multiple gas inlets and designed to work over a wide range of pressures and temperatures. The reaction chamber was loaded with oxygen (2.5 bar) and pressurized with nitrogen (up to 20 bar) [57]. Reactions were performed in the presence of benzylalcohol, K_2_CO_3_ in methanol and numerous different reaction conditions were screened (Table 1).

**Table 1:** Oxidative esterification of benzylalcohol with methanol.

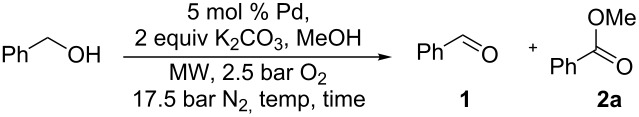					

Entry	Pd catalyst	Temp, Time [°C, h]	Conv [%]	**1** [%]^a^	**2a** [%]^a^

1	PdCl_2_(PPh_3_)_2_	MW, 90 °C, 1 h	35	17	12
2	PdCl_2_(PPh_3_)_2_	MW, 70 °C, 2 h	48	35	11
3	Pd(OAc)_2_	MW, 90 °C, 1 h	100	49	51
4^b^	Pd(OAc)_2_	MW, 90 °C, 1 h	88	46	42
5^c^	Pd(OAc)_2_	MW, 90 °C, 1 h	82	41	36
6	Pd(OAc)_2_	MW, 70 °C, 2 h	80	46	34
7^d^	Pd(OAc)_2_	MW, 90 °C, 1 h	100	0	100
8	Pd(OAc)_2_/C	MW, 90 °C, 1 h	100	11	89
9	10% Pd/C	MW, 90 °C, 1 h	100	0	100
10^d^	10% Pd/C	MW, 90 °C, 1 h	100	0	100
11	10% Pd/C	MW, 70 °C, 2 h	100	0	100
12^d^	Pd(PPh_3_)_4_	MW, 90 °C, 1 h	2.3	2	–
13^d,e^	10% Pd/C	60 °C, 22 h	2.5	3	0
14^d,f^	10% Pd/C	90 °C, 3.5 h	1.2	1	–
15^d,f^	10% Pd/C	90 °C, 7 h	15	15	0

^a^Isolated yield. ^b^The reaction was performed with 10 mol % Pd. ^c^10 mol % triphenylphosphine was added. ^d^The mixture of the base in 1 mL MeOH was sonicated in an ultrasound bath for 10 s prior to the addition of the catalyst and the substrate. ^e^O_2_ balloon. ^f^The reaction was performed in Parr 2.5 bar O_2_/17.5 bar N_2_.

As depicted in Table 1, Pd(OAc)_2_ afforded the methyl ester (selectivity 51%) after 1 h MW irradiation at 90 ^o^C (Table 1, entry 3) and the optimization of the process was pursued. It is noteworthy that longer reaction times at a lower temperature (Table 1, entry 6), doubling the amount of catalyst (Table 1, entry 4) or the addition of an electron-rich ligand such as triphenylphosphine to the reaction mixture (Table 1, entry 5) only had minimal effects on substrate conversion and selectivity. Preliminary sonication of the K_2_CO_3_ in methanol, prior to adding the catalyst and the substrate, led to a significant increase in reactivity affording complete conversion and complete selectivity (compare Table 1, entries 3 and 7). Because of the excellent dielectric properties of charcoal, we tested activated charcoal adsorbed Pd(OAc)_2_ and observed complete conversion and good selectivity (Table 1, entry 8). Based on this result and on a recently published study by Stahl et al. [40], it was decided that the investigation be broadened to the readily accessible Pd/C. Gratifyingly, full substrate conversion to the desired ester was afforded without catalyst pre-sonication, even at 70 °C (Table 1, entries 9–11). For the sake of comparison, the oxidative esterification of benzylalcohol in the presence of methanol was attempted under conventional heating. Reactions were performed under O_2_ pressure (simple rubber balloon and Parr reactor at 2.5 bar) at 60 °C and 90 °C (Table 1, entry 13–15). Poor conversion was detected at low O_2_ pressure at 60 °C after 22 h and yields were not improved in the Parr reactor. The optimized protocol under MW was repeated using the Parr reactor filled with O_2_ (2.5 bar) and pressurized with N_2_ (17.5 bar). The low value of 15% conversion was observed after 7 h stirring at 90 °C.

These encouraging results prompted us to screen for the influence of different bases on reaction conversion and selectivity when the reaction was catalyzed by Pd(OAc)_2_, Pd(OAc)_2_/C, and Pd/C.

**Figure 1:**
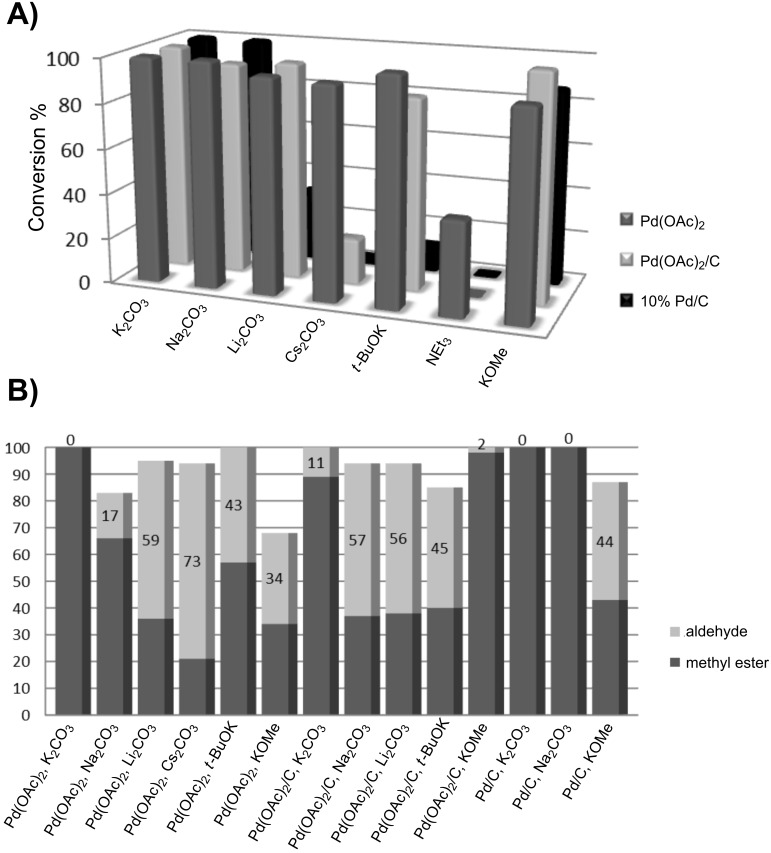
Screening of different catalysts and bases in the catalytic oxidative esterification of benzylalcohol. A) Comparison of the methyl benzoate yield. B) Comparison of the benzylalcohol conversion and selectivity. Conditions: 1 mmol benzylalcohol, 5 mol % Pd, 2 equiv base, 1 mL methanol, O_2_ (2.5 bar), N_2_ 17.5 bar.

The three-dimensional array in Figure 1A highlights that high conversion was generally achieved under different conditions. Good results were obtained with Pd (OAc)_2_ under homogenous and heterogeneous catalysis. The reactivity of Pd/C was observed to be more influenced by the kind of base and only K_2_CO_3,_ Na_2_CO_3_ and KOMe gave good conversion (see also Supporting Information File 1). The selectivity of experiments that gave the highest conversions was compared in the two-dimensional array (Figure 1B). As depicted, Pd/C gave complete selectivity in the presence of either Na_2_CO_3_ or K_2_CO_3_, while Pd(OAc)_2_ was much more selective in the presence of K_2_CO_3_ when the reaction was performed in the homogeneous phase. Charcoal supported Pd(OAc)_2_ showed the highest selectivity with KOMe.

As depicted in Table 2, the optimized Pd/C catalyzed oxidative esterification protocol exhibits general applicability with various aldehydes. High methyl ester yields were obtained in most cases. The catalytic process proved to be substrate dependent to an extent, so an appropriate reaction temperature, ranging from 90 to 120 °C, had to be applied for each substrate. Thiophene carboxaldehyde, for instance, afforded only a 45% desired ester yield at 100 °C, while the yield increased to 85% in the absence of any byproducts when the reaction temperature was raised to 120 °C (Table 2, entry 12). However, significant amounts of decarbonylated analogues were detected when the reaction was performed at 120 °C with the nitrobenzaldehyde derivatives (Table 2, entries 4 and 5). The aldehyde decarbonylation reaction in Pd catalytic systems has recently been reported in the literature [58–59]. High conversion and selectivity were achieved in most cases, apart from 3,5-dimethoxybenzaldehyde and 4-chlorobenzaldehyde.

**Table 2:** Oxidative esterification of various aldehydes in the presence of methanol.

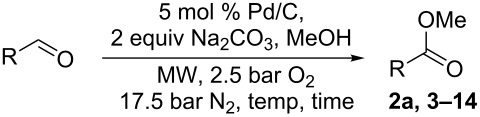				

Entry	Substrate	Temp, Time [°C, h]	Conv. [%]^a^	Ester [%]^a^

1	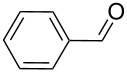	100, 1	100	98
2	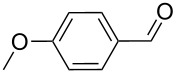	90, 2	100	98
3	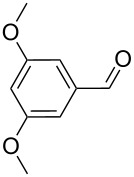	100, 1	75	50
4	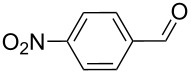	100, 1	100	94^b^
5	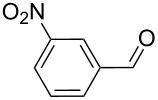	90, 2	100	98^c^
6	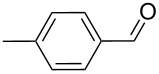	100, 1	100	98
7	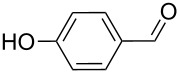	100, 1	100	96
8	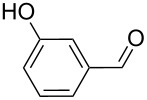	100, 1	100	98
9	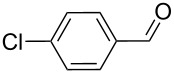	100, 1	23	13
10	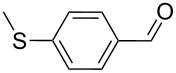	120, 1.5	85	63
11	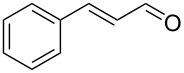	120, 1.5	65	70
12	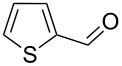	120, 1.5	85	73
13	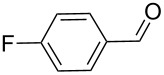	120, 1.5	100	97

^a^Isolated yield; ^b^when repeated at 120 °C (1,5 h) 15% of nitrobenzene was detected by GC–MS; ^c^when repeated at 120 °C (1,5 h) 22% of nitro benzene was detected by GC–MS.

Pd-catalyzed dehalogenation reaction of 4-chlorobenzaldehyde (120 °C for 1.5 h) gave methyl benzoate as main product besides traces of the desired product (detected by GC–MS analysis). Aiming to reduce this undesired reaction, the temperature was decreased to 100 °C, in this way, despite the low conversion, 13% of product was obtained.

Table 3 depicts the results of the oxidative esterification, performed with a series of alcohols. In most cases, the substrates were quantitatively oxidized to the corresponding methyl esters within 1–2 hours. Reaction conditions and temperature were optimized for each substrate. The oxidative esterification of benzylalcohol in the presence of aliphatic alcohols other than methanol was then studied (Table 3). As depicted in Table 3 entry 1 complete conversion was achieved with high selectivity for the corresponding esters of benzylalcohol with ethanol, *n*-PrOH, iPrOH and *n*-BuOH. When the reaction was performed with *n*-BuOH we observed the presence of butyl butyrate.

**Table 3:** Oxidative esterification of various alcohols.

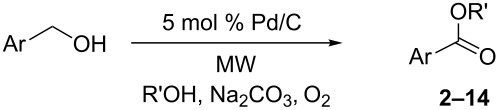				

Entry	Substrate	R’	Temp. [°C], Time [h]	Yield [%]^a^

1	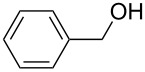	Me Et *n-*Pr iPr *n-*Bu	100, 1 120, 1.5 120, 1.5 120, 1.5 120, 1.5	98 98 79 78 76
2	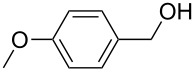	Me	120, 1.5	98
3	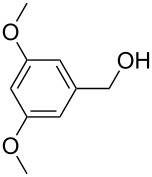	Me	120, 1.5	36
4	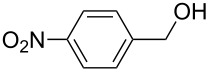	Me	90, 2	44
5	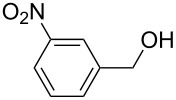	Me	100, 2	58
6	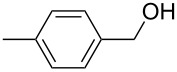	Me	120, 1.5	97
7	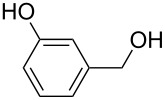	Me	120, 1.5	89
8	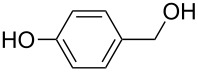	Me	120, 1.5	96
9	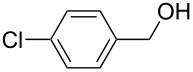	Me	100, 1	8
10	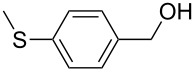	Me	120, 1.5	85
11	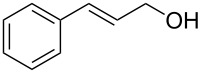	Me	120, 1.5	35
12	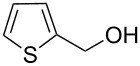	Me	120, 1.5	75
13	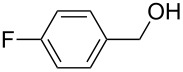	Me	120, 1.5	29

^a^Isolated yield.

When the reaction was performed with benzylalcohol in *n*-BuOH (Table 3, entry 1) the main product detected by GC–MS was the butyl butyrate. Esterification reactions using other linear alcohols as substrate, such as pentanol and hexadecanol carried out in methanol, gave in both cases less than 10% yield of the corresponding esters (data not shown).

**Scheme 2:**
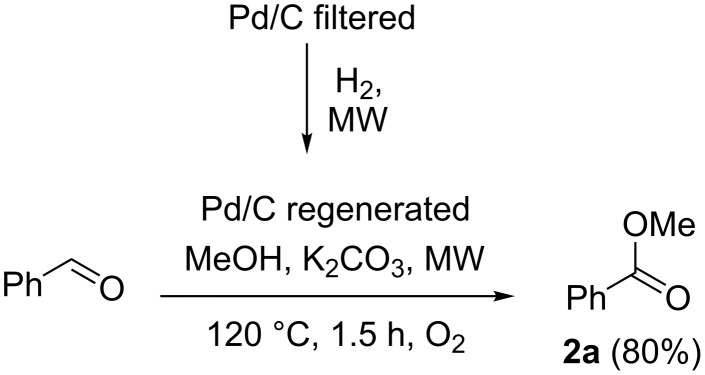
Catalyst regeneration and oxidative esterification of benzaldehyde (2^nd^ cycle).

The recycling of the catalyst was evaluated in order to better investigate and optimize the protocol. The catalyst was filtered on sintered glass and washed three times with methanol. The recovered Pd/C was reused in the esterification of benzaldehyde and benzylalcohol, however only starting material was detected after MW irradiation at 120 °C for 1.5 h. Notably, the Pd(0) species can be oxidized to Pd-peroxo complexes [60]. The peroxo complexes can be reduced back to Pd(0) using hydrogen, therefore we decided to regenerate the catalyst by hydrogenation. As depicted in the Scheme 2, to our delight, the oxidative esterification of benzaldehyde gave methyl benzoate in 80% yield. Based on this evidence, we can assume that Pd(0) can be oxidized by oxygen to obtain unreactive species. Based on Beller at al. [31] the Pd-peroxo complex does not catalyze the oxidative esterification of benzylalcohol, but the mechanism for the Pd catalysed aerobic oxidative esterification has not still been fully elucidated. Additional mechanistic studies will be needed to support a mechanistic rationale.

## Conclusion

In summary, we have described a highly efficient, versatile and eco-friendly protocol for the aerobic esterification of benzylic alcohols and aldehydes to methyl esters. 5 mol % Pd/C under MW irradiation has proven itself to be an effective catalytic system. The reaction proceeded with high conversion and selectivity without the addition of ligands and organic hydrogen acceptors. MW irradiation promoted the reaction and reaction time was dramatically cut. A preliminary evaluation of catalyst recycling showed that Pd/C can be reused for the esterification of benzaldehyde after hydrogenation.

## Experimental

### General methods

All commercially available reagents and solvents were used without further purification. Pd sources from Sigma-Aldrich included 10 wt % Pd/C (Cat. No.75990) and Pd(OAc)_2_ and PdCl_2_(PPh_3_)_2_ from Merck. NMR spectra were recorded with a Bruker 300 Avance (300 MHz and 75 MHz for ^1^H and ^13^C, respectively) at 25 °C. Chemical shifts were calibrated to the residual proton and carbon resonances of the solvent; CDCl_3_ (δH = 7.26, δC = 77.16). GC–MS analyses were performed in a GC Agilent 6850 (Agilent Technologies, Palo Alto, CA, USA) that was fitted with a mass detector Agilent Network 5973, using a 30 m long capillary column HP 5-MS (5% phenyl methyl siloxane, i.d. 0.25 mm, film thickness 0.25 μm). GC conditions were: injection split 1:20, injector temperature 250 °C, detector temperature 280 °C. Gas carrier: helium (1.2 mL/min), temperature program: from 50 °C (held for 3 min) to 80 °C at 3 °C/min and to 300 °C at 10 °C/min. Reactions were carried out in a professional MW reactor SynthWave (MLS GmbH, Milestone S.r.l.). The sonochemical device was developed in collaboration with Danacamerini sas (Torino, Italy).

#### General procedure for aerobic aldehyde and alcohol esterification

Na_2_CO_3_ (2 equiv) was dissolved in MeOH (1 mL) and sonicated with a US bath for 10 sec (20.3 kHz, 60 W). The substrate (aldehyde or alcohol, 1 mmol) and 10% Pd/C (5% Pd/mol of substrate) were added to this mixture. The reaction was carried out under magnetic stirring in a MW reactor SynthWave. The 1 L pressure-resistant PTFE cavity (up to 200 bar) equipped with a 15 position vial rack was loaded with O_2_ (2.5 bar) followed by the addition of N_2_ up to 20 bar total pressure. The reaction was irradiated for an appropriate reaction temperature ranging from 90 to 120 °C (average power 300 W), and for 1 to 2 hours (see Table 2 and Table 3). The mixture was then filtered off through celite, the catalyst washed with MeOH and the solvent evaporated under vacuum. Isolated yields for all substrates reported were obtained using these conditions.

#### General procedure for Pd/C catalyst regeneration

Pd/C, filtered after a first cycle of oxidative esterification of benzylalcohol, was regenerated. 50 mg were dispersed in toluene (1.45 mL). The reaction was carried out under magnetic stirring in the MW reactor (SynthWave). The reactor was loaded with H_2_ (6 bar) pressure followed by the addition of N_2_ up to 20 bar total pressure. The reaction was left at 60 °C for 8 h (average power 150 W). The mixture was then filtered and the catalyst was recovered (0.042 g).

## Supporting Information

File 1 Copies of GC–MS chromatograms, MS, ^1^H NMR and ^13^C NMR spectra.

## References

[1] Mallat T, Baiker A (2004). Chem Rev.

[2] Matsumoto T, Ueno M, Wang N, Kobayashi S (2008). Chem–Asian J.

[3] Davis S E, Ide M S, Davis R J (2013). Green Chem.

[4] Karimia B, Esfahani F K (2012). Adv Synth Catal.

[5] Su F-Z, Liu Y-M, Wang L-C, Cao Y, He H-Y, Fan K-N (2007). Angew Chem, Int Ed.

[6] Mitsudome T, Noujima A, Mizugaki T, Jitsukawa K, Kaneda K (2009). Adv Synth Catal.

[7] Lucchesi C, Inasaki T, Miyamura H, Matsubara R, Kobayashi S (2008). Adv Synth Catal.

[8] Mahyari M, Shaabani A, Bide Y (2013). RSC Adv.

[9] Mori K, Hara T, Mizugaki T, Ebitani K, Kaneda K (2004). J Am Chem Soc.

[10] Karimi B, Abedi S, Clark J H, Budarin V (2006). Angew Chem, Int Ed.

[11] Dun R, Wang X, Tan M, Huang Z, Huang X, Ding W, Lu X (2013). ACS Catal.

[12] Chen G, Wu S, Liu H, Jiang H, Li Y (2013). Green Chem.

[13] Yamaguchi K, Mori K, Mizugaki T, Ebitani K, Kaneda K (2000). J Am Chem Soc.

[14] Yamaguchi K, Mizuno N (2002). Angew Chem, Int Ed.

[15] Yu H, Wu Y, Peng F, Zhang Y, Wang H, Yang J (2012). Catal Lett.

[16] Costa V V, Jacinto M J, Rossi L R, Landers R, Gusevskaya E V (2011). J Catal.

[17] Shiraishi Y, Sakamoto H, Sugano Y, Ichikawa S, Hirai T (2013). ACS Nano.

[18] Suvendu S, Das S, Partha Kumar S, Supriya D, Papu B (2013). RSC Adv.

[19] Chen L, Hu J, Sankar Mal S, Kortz U, Jaensch H, Mathys G, Richards R M (2009). Chem–Eur J.

[20] Dobereiner G E, Crabtree R H (2010). Chem Rev.

[21] Oliveira R L, Kiyohara P K, Rossi L M (2009). Green Chem.

[22] Miyamura H, Yasukawa T, Kobayashi V (2010). Green Chem.

[23] Su F-Z, Ni J, Sun H, Cao Y, He H-Y, Fan K-N (2008). Chem–Eur J.

[24] Ishida T, Nagaoka M, Akita T, Haruta M (2008). Chem–Eur J.

[25] Kaizuka K, Miyamura H, Kobayashi S (2010). J Am Chem Soc.

[26] Tang L, Guo X, Li Y, Zhang S, Zha Z, Wang Z (2013). Chem Commun.

[27] Zhang J, Leitus G, Ben-David Y, Milstein D (2005). J Am Chem Soc.

[28] Owston N A, Parkerb A J, Williams J M J (2008). Chem Commun.

[29] Zhang D, Pan C (2012). Catal Commun.

[30] Yamamoto N, Obora Y, Ishii Y (2011). J Org Chem.

[31] Gowrisankar S, Neumann H, Beller M (2011). Angew Chem, Int Ed.

[32] Liu C, Wang J, Meng L, Deng Y, Li Y, Lei A (2011). Angew Chem, Int Ed.

[33] Bai X-F, Ye F, Zheng L-S, Lai G-Q, Xia C-G, Xu L-W (2012). Chem Commun.

[34] Liu C, Tang S, Lei A (2013). Chem Commun.

[35] Luo F, Pan C, Cheng J, Chen F (2011). Tetrahedron.

[36] Sheldon R A, Arends I W C E, ten Brink G-J, Dijksman A (2002). Acc Chem Res.

[37] Parmeggiani C, Cardona F (2012). Green Chem.

[38] Stahl S S (2004). Angew Chem, Int Ed.

[39] Muzart J (2003). Tetrahedron.

[40] Powell A B, Stahl S S (2013). Org Lett.

[41] Jagadeesh R V, Junge H, Pohl M-M, Radnik J, Brückner A, Beller M (2013). J Am Chem Soc.

[42] Wang N, Matsumoto T, Ueno M, Miyamura H, Kobayashi S (2009). Angew Chem, Int Ed.

[43] Korovchenko P, Donze C, Gallezot P, Besson M (2007). Catal Today.

[44] Heropoulos G A, Villalonga-Barber C (2011). Tetrahedron Lett.

[45] Muzart A, Zeltner M, Stark W J (2012). ACS Catal.

[46] Cravotto G, Beggiato M, Palmisano G, Penoni A, Lévêque J-M, Bonrath W (2005). Tetrahedron Lett.

[47] Tagliapietra S, Cravotto G, Calcio Gaudino E, Visentin S, Mussi V (2012). Synlett.

[48] De La Hoz A, Loupy A (2012). Microwaves in Organic Synthesis.

[49] Barge A, Tagliapietra S, Tei L, Cintas P, Cravotto G (2008). Curr Org Chem.

[50] Palmisano G, Bonrath W, Boffa L, Garella D, Barge A, Cravotto G (2007). Adv Synth Catal.

[51] Kaval N, Dehaen W, Kappe O C, Van der Eycken E (2004). Org Biomol Chem.

[52] Will H, Scholz P, Ondruschka B (2002). Chem Ing Tech.

[53] Tanner D D, Kandanarachchi P, Ding Q, Shao Q, Vizitiu D, Franz J A (2001). Energy Fuels.

[54] Zhang X, Lee C S-M, Mingos D M P, Hayward D O (2003). Catal Lett.

[55] Zhang X, Hayward D O, Mingos D M P (2003). Catal Lett.

[56] Carnaroglio D, Martina K, Palmisano G, Penoni A, Domini C, Cravotto G (2013). Beilstein J Org Chem.

[R57] 57The reactions were performed in small scale. 17.5 bar of N_2_ were added to increase the total pressure of the system. In this condition, the pressure of O_2_ was 2.5 bar and the flammability hazard was limited. Working at 20 bar of total pressure, the reactions were heated over the boiling point of the solvent.

[58] Modak A, Deb A, Patra T, Rana S, Maity S, Maiti D (2012). Chem Commun.

[59] Akanksha, Maiti D (2012). Green Chem.

[60] Sergeev A G, Neumann H, Spannenberg A, Beller M (2010). Organometallics.

