# Supplementary material for: Pd/C-catalyzed aerobic oxidative esteriﬁcation of alcohols and aldehydes: a highly efficient microwave-assisted green protocol
Source: Beilstein J Org Chem. 2014 Jun 26;10:1454–61. doi: 10.3762/bjoc.10.149 (PMC4077537; doi:10.3762/bjoc.10.149)
Supplement: File 1 — Copies of GC–MS chromatograms, MS, 1H NMR and 13C NMR spectra. [file Beilstein_J_Org_Chem-10-1454-s001.pdf]

Supporting Information

for

Pd/C-catalyzed aerobic oxidative esterification of alcohols and aldehydes: a highly efficient microwave-assisted green protocol

Marina Caporaso[1], Giancarlo Cravotto*[1,2], Spyros Georgakopoulos[1,3], George Heropoulos[3], Katia Martina[1] and Silvia Tagliapietra[1]

Address: [1]Dipartimento di Scienza e Tecnologia del Farmaco, University of Turin, Via P. Giuria 9, 10125 Torino, Italy. Fax +390116707687; Tel: +390116707684, [2]Interdepartmental Centre "Nanostructured interfaces and surfaces- NIS, University of Turin, Via P. Giuria 7-9, 10125 Torino, Italy and [3]Institute of Biology, Medicinal Chemistry and Biotechnology, National Hellenic Research Foundation, 48. Vas. Constantinou Ave., 11635 Athens, Greece.

Email: Giancarlo Cravotto - giancarlo.cravotto@unito.it

*Corresponding author

Authors are listed in alphabetical order and contributed equally to this work.

Copies of GC–MS chromatograms, MS, ^{1}H NMR and ^{13}C NMR spectra

Table of Contents:

General experimental methods S2

General procedure for Pd/C catalyst regeneration S2

General procedure for the synthesis of Pd(OAc)2/C catalyst S2

Table S1: Oxidative esterification of benzylalcohol in the presence of methanol over different bases S3

Compound characterization S4–S10

GC–MS analysis chromatograms of crude reactions S11–S27

References S28

General experimental methods

All commercially available reagents and solvents were used without further purification. Pd sources from Sigma-Aldrich included 10 wt % Pd/C (Cat. No.75990) and $Pd(OAc)_2$ and $PdCl_2(PPh_3)_2$ from Merck.

NMR spectra were recorded with a Bruker 300 Avance (300 MHz and 75 MHz for ^1H and ^{13}C, respectively) at 25 °C. Chemical shifts were calibrated to the residual proton and carbon resonances of the solvent; $CDCl_3$ (δH = 7.26, δC = 77.16).

GC–MS analyses were performed in a GC Agilent 6850 (Agilent Technologies, Palo Alto, CA, USA) that was fitted with a mass detector Agilent Network 5973, using a 30 m long capillary column HP 5-MS (5% phenyl methyl siloxane, i.d. 0.25 mm, film thickness 0.25 μm). GC conditions were: injection split 1:20, injector temperature 250 °C, detector temperature 280 °C. Carrier gas: helium (1.2 mL/min), temperature program: from 50 °C (held for 3 min) to 80 °C at 3 °C/min and to 300 °C at 10 °C/min.

Reactions were carried out in a professional MW reactor SynthWave (MLS GmbH, Milestone S.r.l.). This device enables high power density (1.5 kW/L) and inert barosphere and the possibility of simultaneously carrying out multiple reactions with gas insertion.

The sonochemical device was developed in collaboration with Danacamerini sas (Torino, Italy).

General procedure for Pd/C catalyst regeneration

Pd/C filtered after a first cycle of oxidative esterification of benzylalcohol was regenerated. 50 mg were dispersed in toluene (1.45 mL). The reaction was carried out under magnetic stirring in a MW reactor SynthWave. The reactor was loaded with H_2 (6 bar) pressure followed by addition of N_2 up to 20 bar total pressure. The reaction was left at 60 °C for 8 hours (average power 150 W). The mixture was then filtered and the catalyst was recovered (0.042g).

General procedure for the synthesis of $Pd(OAc)_2$/C catalyst

Activated charcoal (0.200 g) was dispersed in MeOH (5 mL) and a solution of $Pd(OAc)_2$ (0.01 g) in MeOH (2.5 mL) was added to dropwise.

The reaction mixture was stirred at rt for 4 hours until the reaction mixture was colorless. The mixture was then filtered and washed with MeOH. The supported catalyst $Pd(OAc)_2$/C was recovered (0.21 g).

Table S1: Oxidative esterification of benzylalcohol in the presence of methanol over different bases.

Entry	Pd catalyst	Base	Conversion [%]	**1**[a] [%]	**2**[a] [%]
1[a]	$Pd(OAc)_2$	K_2CO_3	100	–	100
2[a]	$Pd(OAc)_2$	Na_2CO_3	100	17	66
3[a]	$Pd(OAc)_2$	Li_2CO_3	95	59	36
4	$Pd(OAc)_2$	Cs_2CO_3	94	73	21
5	$Pd(OAc)_2$	*t*-BuOK	100	43	57
6	$Pd(OAc)_2$	NEt_3	42	30	7
7[b]	$Pd(OAc)_2$	KOMe	91	34	55
8[b]	10% Pd/C	K_2CO_3	100	–	100
9[b,c]	10% Pd/C	Na_2CO_3	100	–	100
10[b]	10% Pd/C	Li_2CO_3	32	25	7
11[b]	10% Pd/C	Cs_2CO_3	3	3	–
12[b]	10% Pd/C	*t*-BuOK	12	10	3
13[b]	10% Pd/C	NEt_3	0	–	–
14[b]	10% Pd/C	KOMe	87	44	43
15[d]	5%$Pd(OAc)_2$/C	K_2CO_3	100	11	89
16[d]	5%$Pd(OAc)_2$/C	Na_2CO_3	94	57	37
17[b]	5%$Pd(OAc)_2$/C	Li_2CO_3	96	56	38
18[b]	5%$Pd(OAc)_2$/C	Cs_2CO_3	20	18	2
19[b]	5%$Pd(OAc)_2$/C	*t*-BuOK	85	45	40
20[b]	5%$Pd(OAc)_2$/C	NEt_3	0	–	–
21[b]	5%$Pd(OAc)_2$/C	KOMe	100	2	98

Reaction conditions: 1 mL MeOH, 0.1 g benzylalcohol, 2 equiv base, catalyst (5% Pd/mole of substrate), 2.5 bar O_2/17.5 bar N_2, MW reactor SynthWave, T = 90 °C (unless otherwise noted), 1 h.

[a] Determined by GC–MS.

[b] The mixture of the base in MeOH was sonicated in an US bath for 10 s prior to the addition of the catalyst and the substrate.

[c] T = 100 °C.

[d] 10% Pd/mole of substrate was added.

Compound characterization

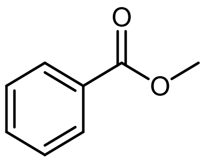

Methyl benzoate (2a)

General procedure for aerobic aldehyde esterification was performed at 100 °C for 1 h with benzaldehyde (0.1 g, 0.942 mmol) to yield the desired ester as a colorless oil (0.126 g, 98%). When synthesized starting from benzylalcohol (0.1 g, 0.925 mmol), the reaction was performed at 100 °C for 1 h to yield the desired ester as a colorless oil (0.124 g, 98%). ^{1}H NMR (300 MHz, $CDCl_3$) and ^{13}C NMR (75 MHz, $CDCl_3$) were in accordance with the reported values in literature[1]. GC–MS (M^+ electron impact), *m/z* calcd for $C_8H_8O_2$ 136.05, found 136, 105, 77, 51; t_R = 14.470 min.

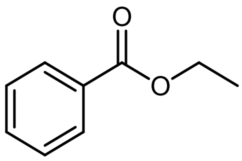

Ethyl benzoate (2b)

General procedure for aerobic alcohol esterification was performed in EtOH at 120 °C for 1.5 h with benzylalcohol (0.1 g, 0.925 mmol) in EtOH to yield the desired ester as a colorless oil (0.136 g, 98%). ^{1}H NMR (300 MHz, $CDCl_3$) and ^{13}C NMR (75 MHz, $CDCl_3$) were in accordance with the reported values in literature[1]. GC–MS (M^+ electron impact), *m/z* calcd for $C_9H_{10}O_2$ 150.07, found 150, 122, 105, 77, 51; t_R = 16.538 min.

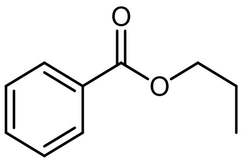

Propyl benzoate (2c)

General procedure for aerobic alcohol esterification was performed in *n*-PrOH at 120 °C for 1.5 h with benzylalcohol (0.1 g, 0.925 mmol). The dried reaction mixture was diluted with CH_2Cl_2, washed with brine and finally dried (anhydrous Na_2SO_4).The crude residue was purified by column chromatography (PE/EtOAc) to yield the desired ester as a colorless oil (0.120 g, 79%). ^{1}H NMR (300 MHz, $CDCl_3$) and ^{13}C NMR (75 MHz, $CDCl_3$) were in accordance with the reported values in literature[2]. GC–MS (M^+ electron impact), *m/z* calcd for $C_{10}H_{12}O_2$ 164.08, found 164, 123, 105, 77, 51; t_R = 18.562 min.

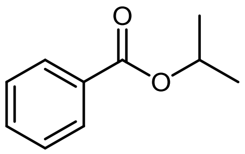

Isopropyl benzoate (2d)

General procedure for aerobic alcohol esterification was performed in iPrOH at 120 °C for 1.5 h with benzylalcohol (0.1 g, 0.925 mmol). The dried reaction mixture was diluted with CH_2Cl_2, washed with brine and finally dried (anhydrous Na_2SO_4).The crude residue was purified by column chromatography (PE/EtOAc) to yield the desired ester as a colorless oil (0.119 g, 78%). ^{1}H NMR (300 MHz, $CDCl_3$) and ^{13}C NMR (75 MHz, $CDCl_3$) were in accordance with the reported values in literature[3]. GC–MS (M^+ electron impact), *m/z* calcd for $C_{10}H_{12}O_2$ 164.08, found 164, 123, 105, 77, 51, 44, 32, 28, 18; t_R = 17.335 min.

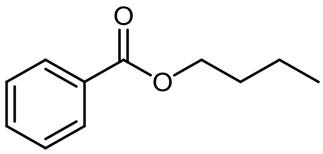

Butyl benzoate (2e)

General procedure for aerobic alcohol esterification was performed in *n*-BuOH at 120 °C for 1.5 h with benzylalcohol (0.1 g, 0.925 mmol). The dried reaction mixture was diluted with CH_2Cl_2, washed with brine and finally dried (anhydrous Na_2SO_4).The crude residue was purified by column chromatography (PE/EtOAc) to yield the desired ester as a colorless oil (0.126 g, 76%). ^{1}H NMR (300 MHz, $CDCl_3$) and ^{13}C NMR (75 MHz, $CDCl_3$) were in accordance with the reported values in literature[4]. GC–MS (M^+ electron impact), *m/z* calcd for $C_{10}H_{14}O_2$ 178.10, found 178, 123, 105, 77, 51; t_R = 20.244 min.

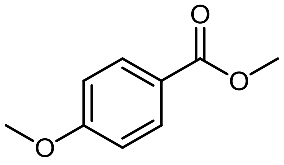

Methyl 4-methoxybenzoate (3)

General procedure for aerobic aldehyde esterification was performed at 90 °C for 2 h with 4-methoxybenzaldehyde (0.1 g, 0.734 mmol) to yield the desired ester as a colorless oil (0.120 g, 98%). When synthesized starting from 4-metyhoxybenzylalcohol (0.1 g, 0.724 mmol), the reaction was performed at 120 °C for 1.5 h to yield the desired ester as a colorless oil (0.124 g, 98%). ^{1}H NMR (300 MHz, $CDCl_3$) and ^{13}C NMR (75 MHz, $CDCl_3$) were in accordance with the reported values in literature[1]. GC–MS (M^+ electron impact), *m/z* calcd for $C_9H_{10}O_3$ 166.06, found 166, 135, 107, 91, 77; t_R = 20.270 min.

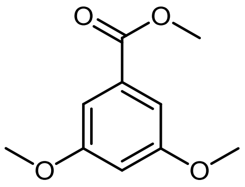

Methyl 3,5-dimethoxybenzoate (4)

General procedure for aerobic aldehyde esterification was performed at 100 °C for 1 h with 3,5-dimethoxybenzaldehyde (0.1 g, 0.602 mmol). The dried reaction mixture was diluted with CH_2Cl_2, washed with brine and finally dried (anhydrous Na_2SO_4).The crude residue was purified by column chromatography (PE/EtOAc) to yield the desired methyl ester as a white powder (0.0354 g, 50%). When synthesized starting from 4-metyhoxybenzylalcohol (0.1 g, 0.724 mmol), the reaction was performed at 120 °C for 1.5 h and the same work-up purification was carried out. The desired ester was obtained as a colorless oil (0.0512 g, 36%). ^{1}H NMR (300 MHz, $CDCl_3$) and ^{13}C NMR (75 MHz, $CDCl_3$) were in accordance with the reported values in literature[5]. GC–MS (M^+ electron impact), *m/z* calcd for $C_{10}H_{12}O_4$ 196.07, found 196, 165, 138, 122, 107, 77, 63; t_R = 22.995 min.

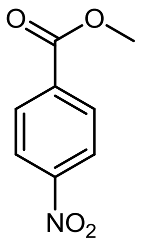

Methyl 4-nitrobenzoate (5)

General procedure for aerobic aldehyde esterification was performed at 100 °C for 1 h with 4-nitrobenzaldehyde (0.1 g, 0.662 mmol). The dried reaction mixture was diluted with CH_2Cl_2, washed with brine and finally dried (anhydrous Na_2SO_4).The crude residue was purified by column chromatography (PE/EtOAc) to yield the desired methyl ester as a white powder (0.113 g, 94%). When synthesized starting from 4-nitrobenzylalcohol (0.1 g, 0.653 mmol), the reaction was performed at 90 °C for 2 h and the same work-up purification was carried out. The desired ester was obtained as a colorless oil (0.0522 g, 44%). ^{1}H NMR (300 MHz, $CDCl_3$) and ^{13}C NMR (75 MHz, $CDCl_3$) were in accordance with the reported values in literature[5]. GC–MS (M^+ electron impact), *m/z* calcd for $C_{10}H_{12}O_4$ 196.07, found 196, 165, 138, 122, 107, 77, 63; t_R = 22.995 min. ^{1}H NMR (300 MHz, $CDCl_3$) and ^{13}C NMR (75 MHz, $CDCl_3$) were in accordance with the reported values in literature[6]. GC–MS (M^+ electron impact), *m/z* calcd for $C_8H_7NO_4$ 181.04, found 181, 164, 150, 104, 92, 76, 50; t_R = 21.497 min.

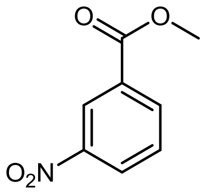

Methyl 3-nitrobenzoate (6)

General procedure for aerobic aldehyde esterification was performed at 90 °C for 2 h with 3-nitrobenzaldehyde (0.1 g, 0.662 mmol) to yield the desired ester as a pale yellow powder (0.118 g, 98%). When synthesized starting from 3-nitrobenzylalcohol (0.1 g, 0.653 mmol), the reaction was performed at 100 °C for 2 h. The dried reaction mixture was diluted with CH_2Cl_2, washed with brine and finally dried (anhydrous Na_2SO_4).The crude residue was purified by column chromatography (PE/EtOAc) to yield the desired methyl ester as a pale yellow powder (0.0686 g, 58%). ^1H NMR (300 MHz, $CDCl_3$) and ^{13}C NMR (75 MHz, $CDCl_3$) were in accordance with the reported values in literature[7]. GC–MS (M^+ electron impact), *m/z* calcd for $C_8H_7NO_4$ 181.04, found 181, 150, 135, 119, 104, 92, 76, 50; t_R = 21.497 min.

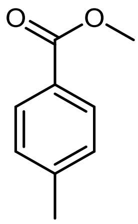

Methyl 4-methylbenzoate (7)

General procedure for aerobic aldehyde esterification was performed at 100 °C for 1 h with 4-methylbenzaldehyde (0.1 g, 0.832 mmol) to yield the desired ester as a colorless oil (0.123 g, 98%). When synthesized starting from 4-methylbenzylalcohol (0.1 g, 0.819 mmol), the reaction was performed at 120 °C for 1.5 h to yield the desired ester as a colorless oil (0.119 g, 97%).
^1H NMR (300 MHz, $CDCl_3$) and ^{13}C NMR (75 MHz, $CDCl_3$) were in accordance with the reported values in literature[1]. GC–MS (M^+ electron impact), *m/z* calcd for $C_9H_{10}O_2$ 150.07, found 150, 119, 91, 65; t_R = 17.458 min.

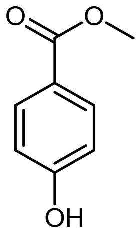

Methyl 4-hydroxybenzoate (8)

General procedure for aerobic aldehyde esterification was performed at 100 °C for 1 h with 4-hydroxybenzaldehyde (0.1 g, 0.819 mmol) to yield the desired ester as a white powder (0.120 g, 96%). When synthesized starting from 4-hydroxybenzylalcohol (0.1 g, 0.806 mmol), the reaction was performed at 120 °C for 1.5 h. The dried reaction mixture was diluted with CH_2Cl_2, washed with brine and finally dried (anhydrous Na_2SO_4).The crude residue was purified by column chromatography (PE/EtOAc) to yield the desired methyl ester as a white powder (0.118 g, 96%). ^1H NMR (300 MHz, $CDCl_3$) and ^{13}C NMR (75 MHz, $CDCl_3$) were in accordance with the reported

values in literature[5]. GC–MS (M^+ electron impact), *m/z* calcd for $C_8H_8O_3$ 152.05, found 152, 121, 93, 65, 39; t_R = 21.024 min.

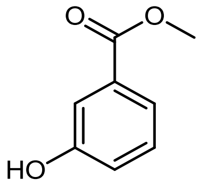

Methyl 3-hydroxybenzoate (9)

General procedure for aerobic aldehyde esterification was performed at 100 °C for 1 h with 3-hydroxybenzaldehyde (0.1 g, 0.819 mmol) to yield the desired ester as a white powder (0.122 g, 98%). When synthesized starting from 3-hydroxybenzylalcohol (0.1 g, 0.806 mmol), the reaction was performed at 120 °C for 1 h. The dried reaction mixture was diluted with CH_2Cl_2, washed with brine and finally dried (anhydrous Na_2SO_4). The crude residue was purified by column chromatography (PE/EtOAc) to yield the desired methyl ester as a white powder (0.110 g, 89%). ^{1}H NMR (300 MHz, $CDCl_3$) and ^{13}C NMR (75 MHz, $CDCl_3$) were in accordance with the reported values in literature[2]. GC–MS (M^+ electron impact), *m/z* calcd for $C_8H_8O_3$ 152.05, found 152, 121, 93, 65, 28, 18; t_R = 21.383 min.

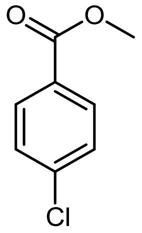

Methyl 4-chlorobenzoate (10)

General procedure for aerobic aldehyde esterification was performed at 100 °C for 1 h with 4-chlorobenzaldehyde (0.1 g, 0.711 mmol). The dried reaction mixture was diluted with CH_2Cl_2, washed with brine and finally dried (anhydrous Na_2SO_4).The crude residue was purified by column chromatography (PE/EtOAc) to yield the desired methyl ester as a colorless oil (0.0157 g, 13%). When synthesized starting from 4-chlorobenzylalcohol (0.1 g, 0.701 mmol), the reaction was performed at 100 °C for 1 h and the same work-up purification was carried out. The desired ester was obtained as a colorless oil (0.00953 g, 8%). ^{1}H NMR (300 MHz, $CDCl_3$) and ^{13}C NMR (75 MHz, $CDCl_3$) were in accordance with the reported values in literature[1]. GC–MS (M^+ electron impact), *m/z* calcd for $C_8H_7ClO_2$ 170.01, found 170, 139, 111, 75; t_R = 18.264 min.

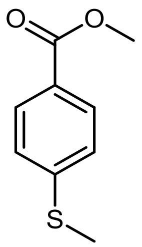

Methyl 4-(methylthio)benzoate (11)

General procedure for aerobic aldehyde esterification was performed at 120 °C for 1.5 h with 4-(methylthio)benzaldehyde (0.1 g, 0.657 mmol). The dried reaction mixture was diluted with CH_2Cl_2, washed with brine and finally dried (anhydrous Na_2SO_4).The crude residue was purified by column chromatography (PE/EtOAc) to yield the desired methyl ester as a white powder (0.0754 g, 63%). When synthesized starting from 4-(methylthio)benzylalcohol (0.1 g, 0.648 mmol), the reaction was performed at 120 °C for 1.5 h and the same work-up purification was carried out. The desired ester was obtained as a white powder (0.100 g, 85%). ^{1}H NMR (300 MHz, $CDCl_3$) and ^{13}C NMR (75 MHz, $CDCl_3$) were in accordance with the reported values in literature[1]. GC–MS (M^+ electron impact), *m/z* calcd for $C_9H_{10}O_2S$ 182.04, found 182, 151, 123, 108; t_R = 22.583 min.

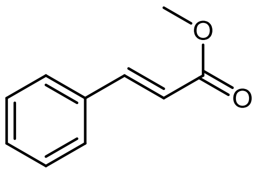

Methyl 3-phenylprop-2-enoate (12)

General procedure for aerobic aldehyde esterification was performed at 120 °C for 1.5 h with 3-phenylprop-2-enal (0.1 g, 0.757 mmol). The dried reaction mixture was diluted with CH_2Cl_2, washed with brine and finally dried (anhydrous Na_2SO_4).The crude residue was purified by column chromatography (PE/EtOAc) to yield the desired methyl ester as a white powder (0.0858 g, 70%). When synthesized starting from 3-phenylprop-2-enol (0.1 g, 0.745 mmol), the reaction was performed at 120 °C for 1.5 h and the same work-up procedure was carried out. The desired ester was obtained as a white powder (0.0422 g, 35%). ^{1}H NMR (300 MHz, $CDCl_3$) and ^{13}C NMR (75 MHz, $CDCl_3$) were in accordance with the reported values in literature[1]. GC–MS (M^+ electron impact), *m/z* calcd for $C_{10}H_{10}O_2$ 162.07, found 162, 131, 103, 77, 51; t_R = 20.323 min.

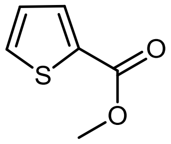

Methyl thiophene-2-carboxylate (13)

General procedure for aerobic aldehyde esterification was performed at 120 °C for 1.5 h with thiophene-2-carbaldehyde (0.1 g, 0.892 mmol). The dried reaction mixture was diluted with CH_2Cl_2, washed with brine and finally dried (anhydrous Na_2SO_4).The crude residue was purified by column chromatography (PE/EtOAc) to yield the desired methyl ester as a colorless oil (0.0925 g, 73%). When synthesized starting from thiophene-2-ylmethanol (0.1 g, 0.876 mmol), the reaction was performed at 120 °C for 1.5 h and the same work-up procedure was carried out. The desired ester was obtained as a colorless oil (0.0933 g, 75%). ^{1}H NMR (300 MHz, $CDCl_3$) and ^{13}C NMR (75 MHz, $CDCl_3$) were in accordance with the reported values in literature[1]. GC–MS (M^+ electron impact), *m/z* calcd for $C_6H_6O_2S$ 142.01, found 142, 111; t_R = 14.829 min.

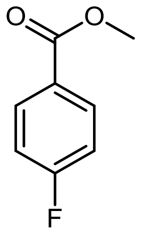

Methyl 4-fluorobenzoate (14)

General procedure for aerobic aldehyde esterification was performed at 120 °C for 1.5 h with 4-fluorobenzaldehyde (0.1 g, 0.806 mmol) to yield the desired ester as a colorless oil (0.121 g, 97%). When synthesized starting from 4-fluorobenzylalcohol (0.1 g, 0.793 mmol), the reaction was performed at 120 °C for 1.5 h. The dried reaction mixture was diluted with CH_2Cl_2, washed with brine and finally dried (anhydrous Na_2SO_4).The crude residue was purified by column chromatography (PE/EtOAc) to yield the desired methyl ester as a colorless oil (0.0355 g, 29%). ^{1}H NMR (300 MHz, $CDCl_3$) and ^{13}C NMR (75 MHz, $CDCl_3$) were in accordance with the reported values in literature[8]. GC–MS (M^+ electron impact), *m/z* calcd for $C_8H_7FO_2$ 154.04, found 154, 123, 95, 75; t_R = 13.734 min.

GC–MS analysis chromatograms of crude reactions

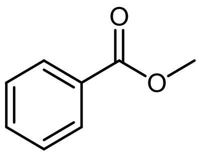

Methyl benzoate (2a)

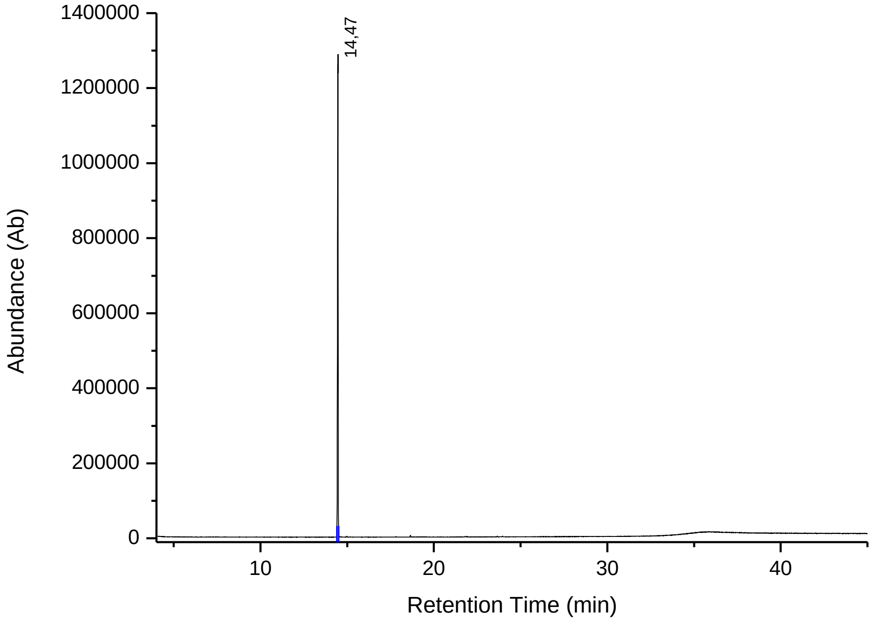

Peak	t_R	Area	% of Total
methyl benzoate	14.470	33436230	100

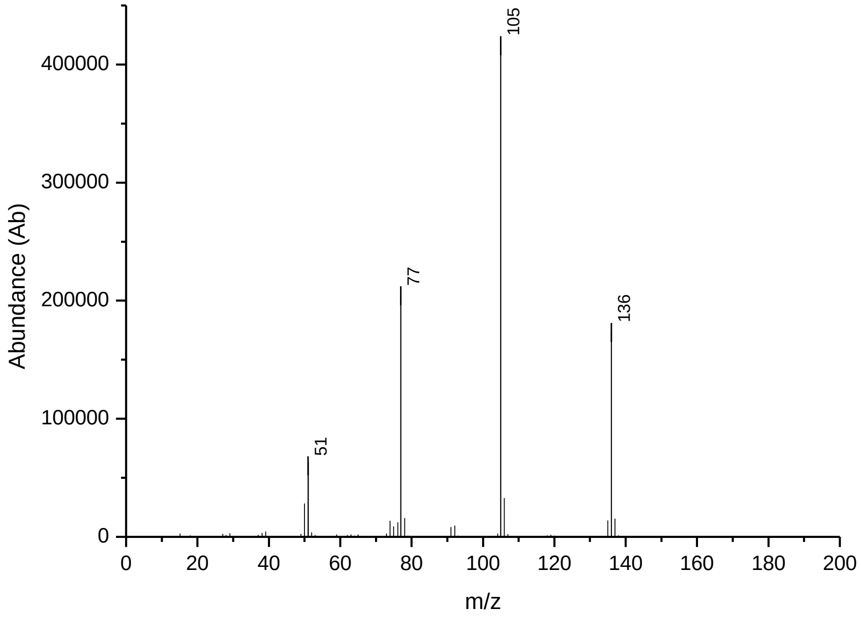

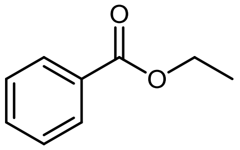

Ethyl benzoate (2b)

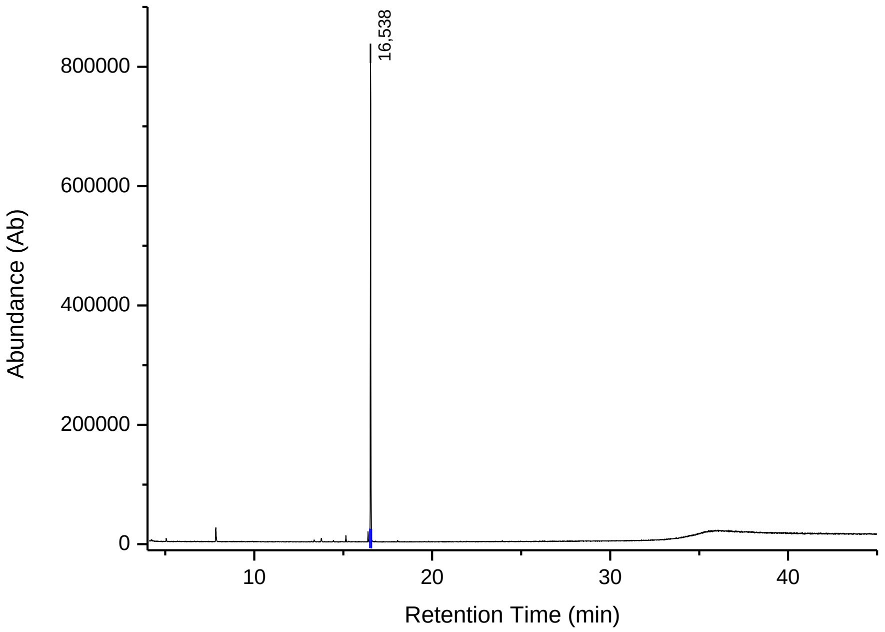

Peak	t_R	Area	% of Total
ethyl benzoate	16.538	14908543	100

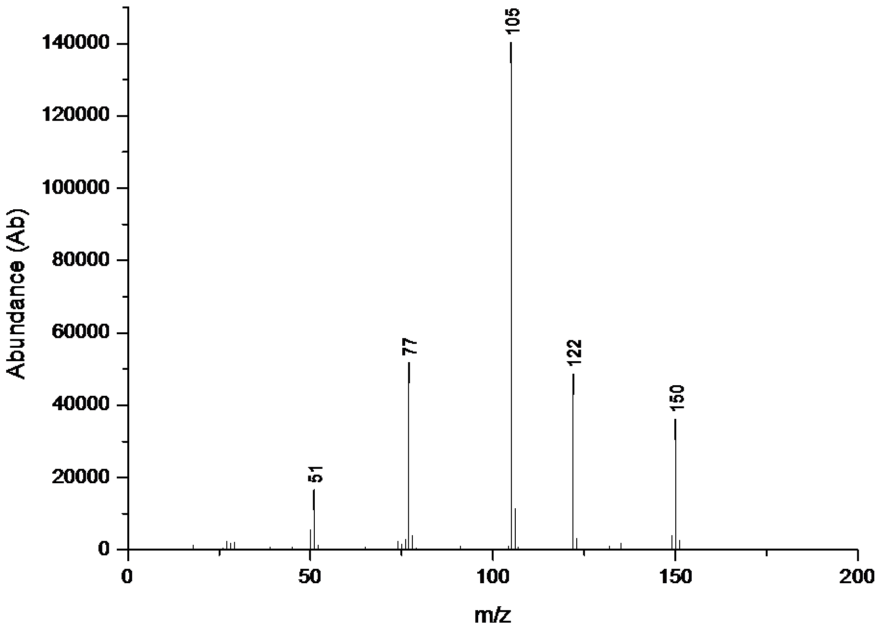

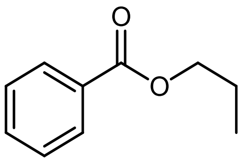

Propyl benzoate (2c)

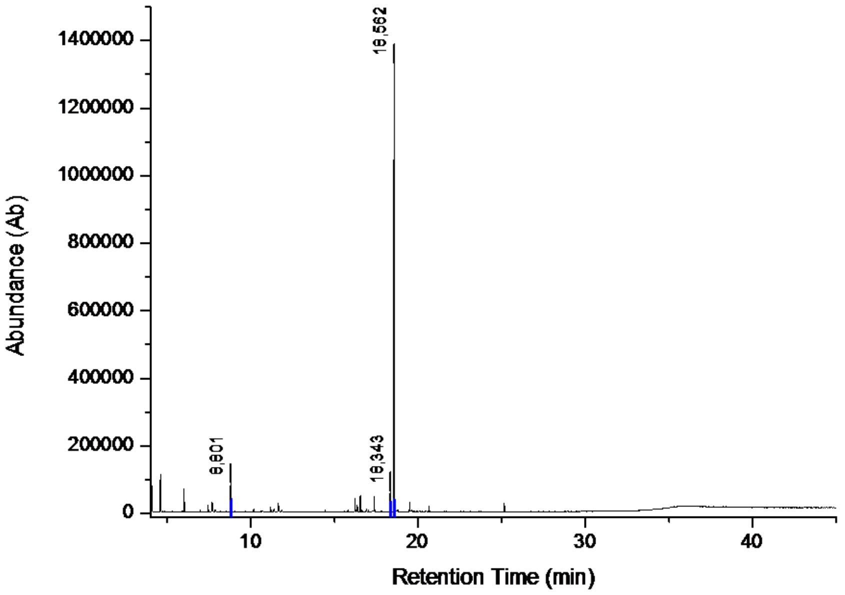

Peak	t_R	Area	% of Total
benzaldehyde	8.801	3072038	12
propanoic acid phenylmethyl ester	18.351	1398640	5
propyl benzoate	18.562	21511949	83

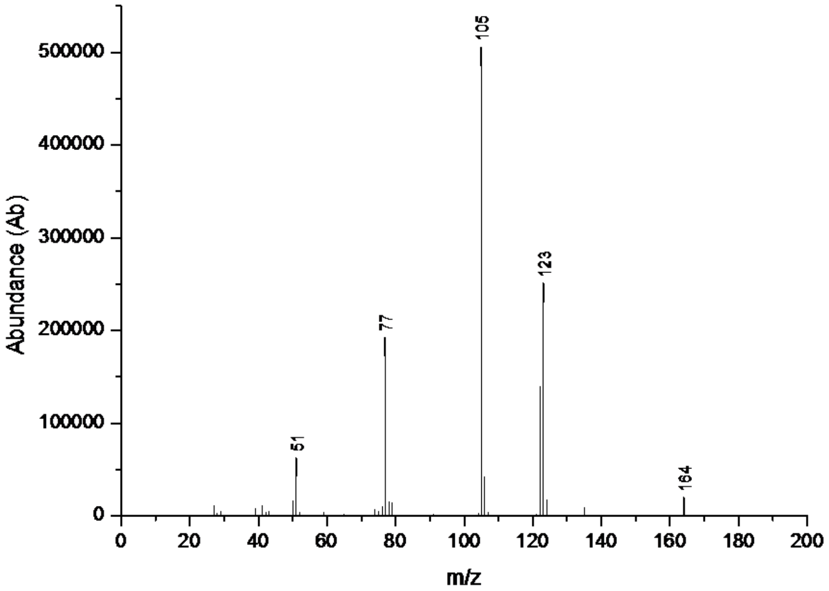

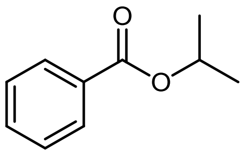

Isopropyl benzoate (2d)

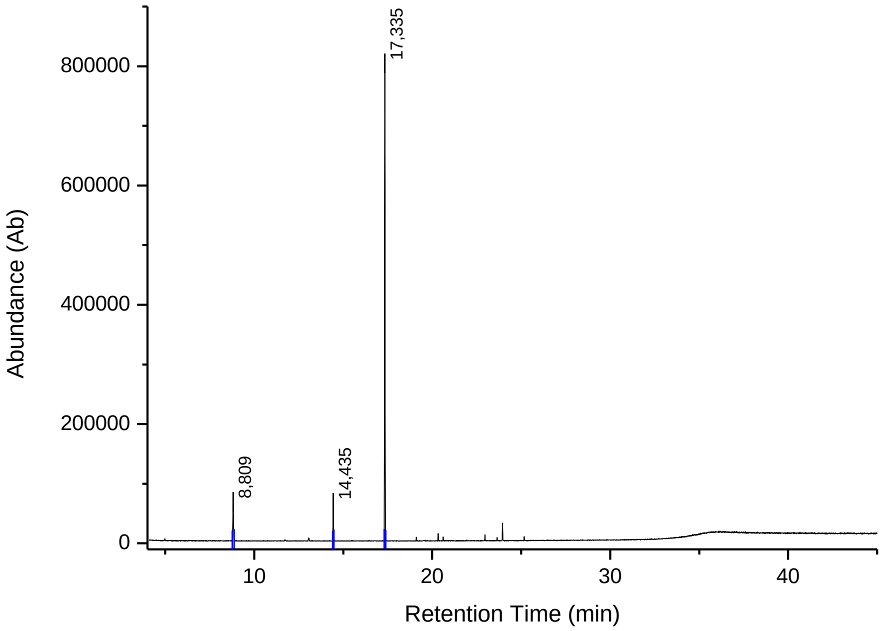

Peak	t_R	Area	% of Total
benzaldehyde	8.773	1781488	11
benzoate	14.416	1526945	9
isopropyl benzoate	17.335	12994139	80

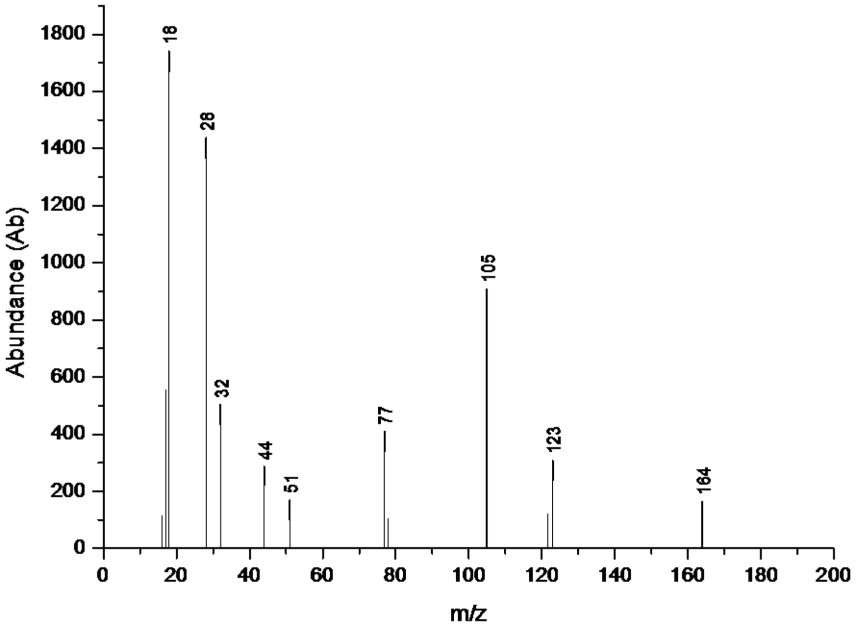

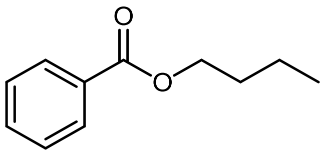

Butyl benzoate (2e)

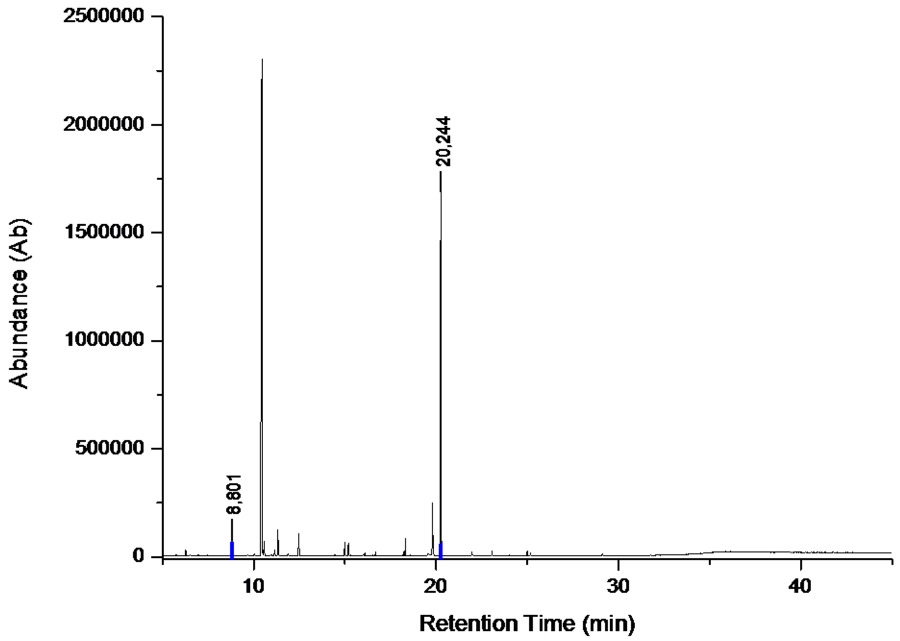

Peak	t_R	Area	% of Total
benzaldehyde	8.801	3368861	9
butyl butyrate	10.448		
butyl benzoate	20.244	28810928	78

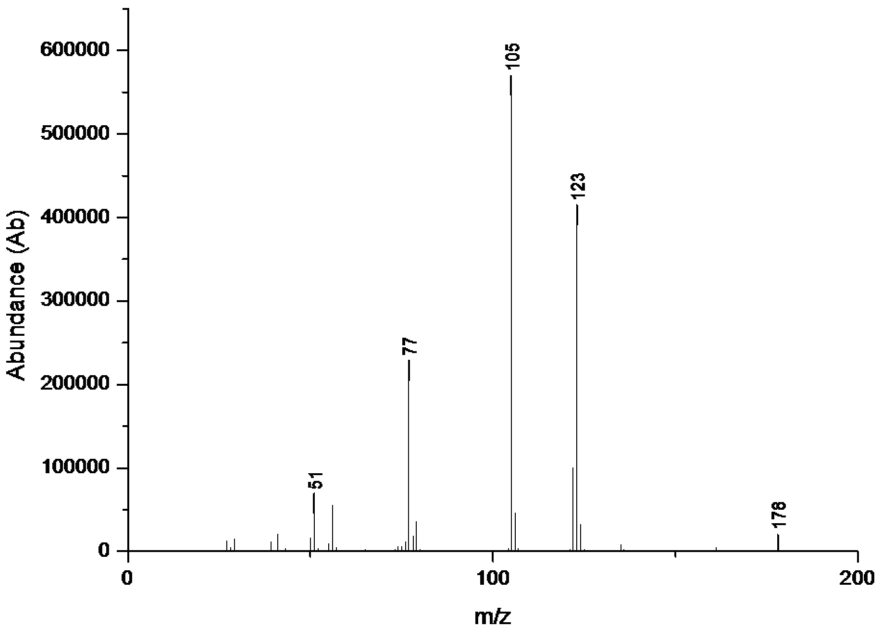

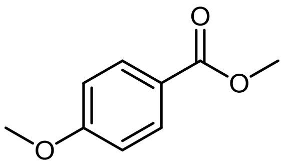

Methyl 4-methoxybenzoate (3)

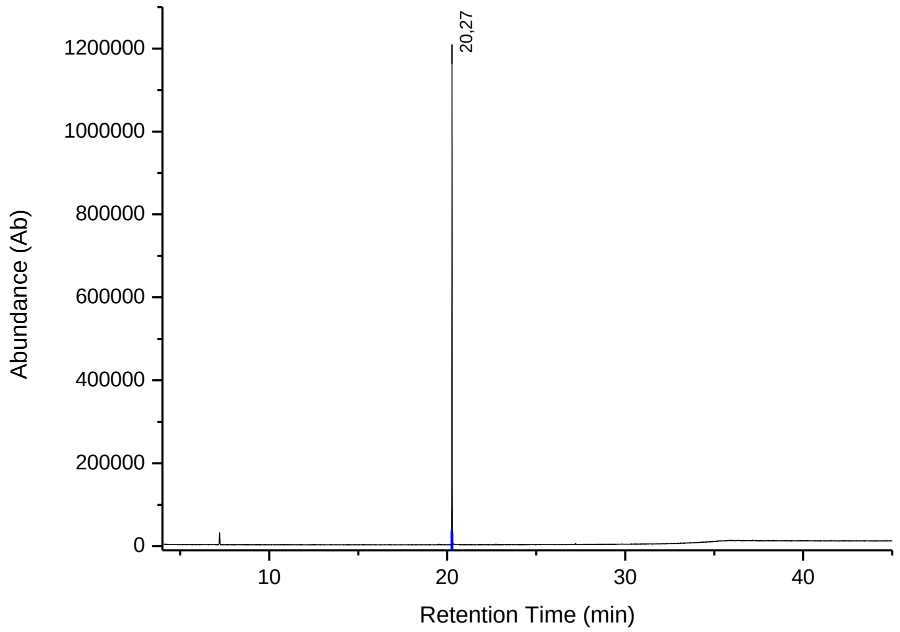

Peak	t_R	Area	% of Total
methyl 3-methoxybenzoate	20.270	18340786	100

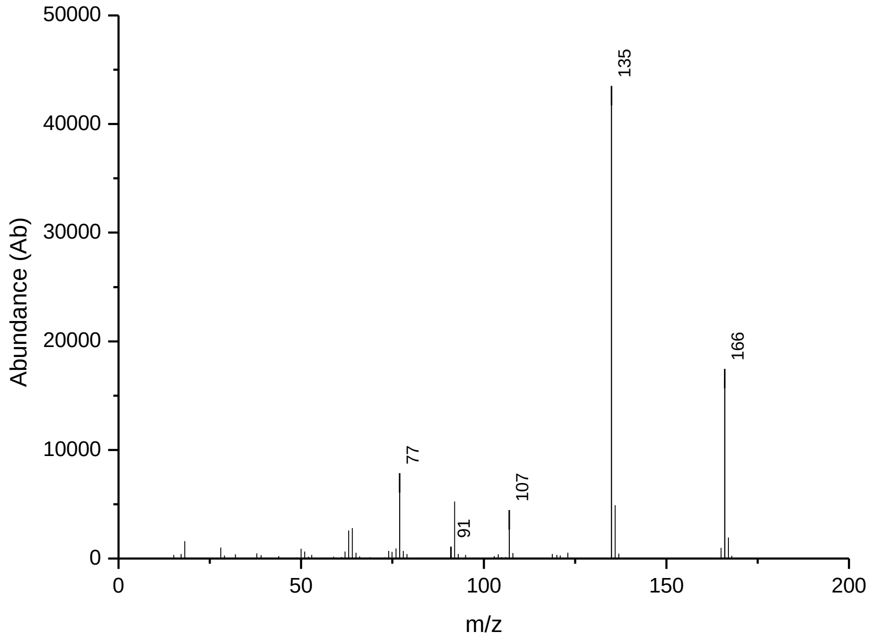

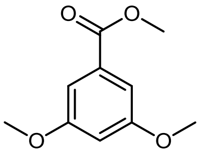

Methyl 3,5-dimethoxybenzoate (4)

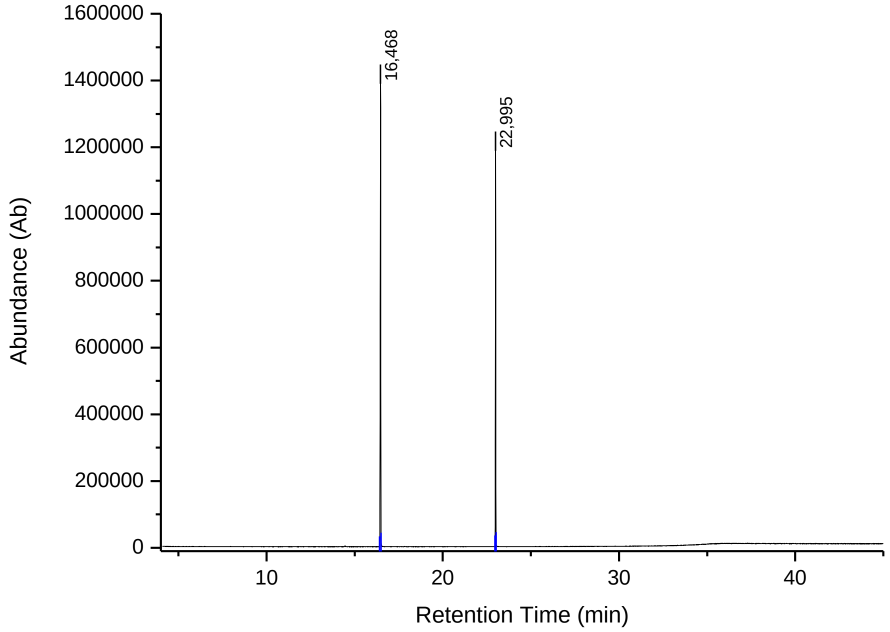

Peak	t_R	Area	% of Total
1,3-dimethoxybenzene	16.468	29952311	62
methyl 3,5-dimethoxybenzoate	22.995	18173718	38

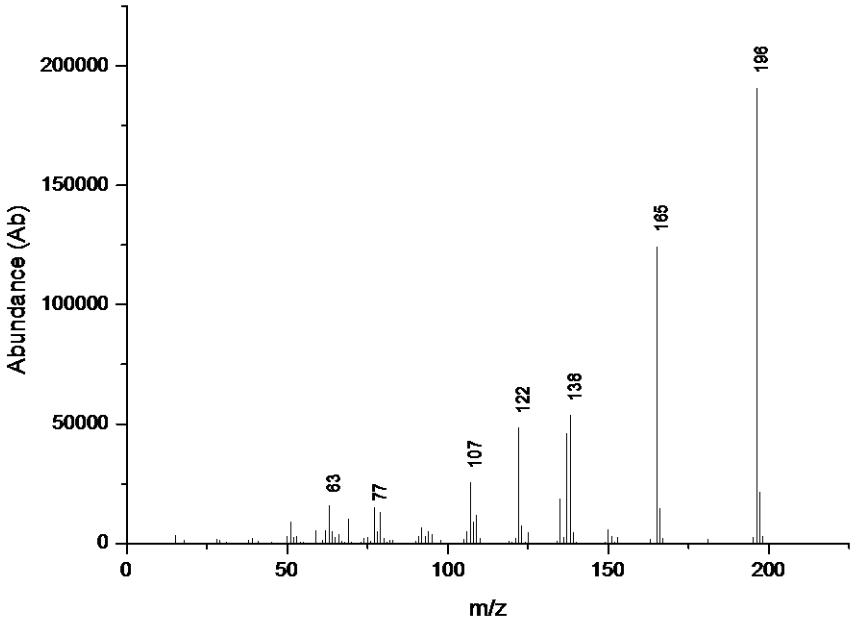

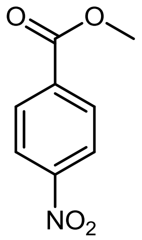

Methyl 4-nitrobenzoate (5)

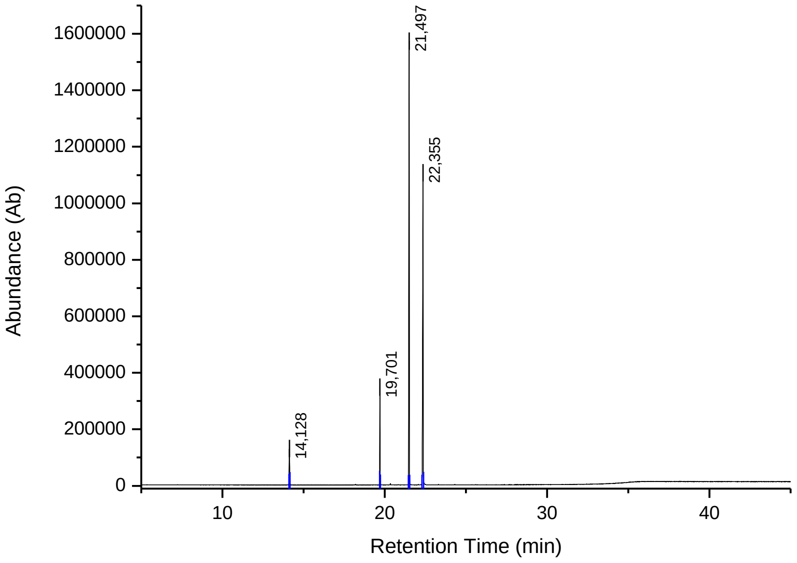

Peak	t_R	Area	% of Total
nitrobenzene	14.128	3172715	5
4-nitrobenzaldehyde	19.701	5144260	8
methyl 4-nitrobenzoate	21.497	29652299	46
4-nitrobenzylalcohol	22.355	26565151	41

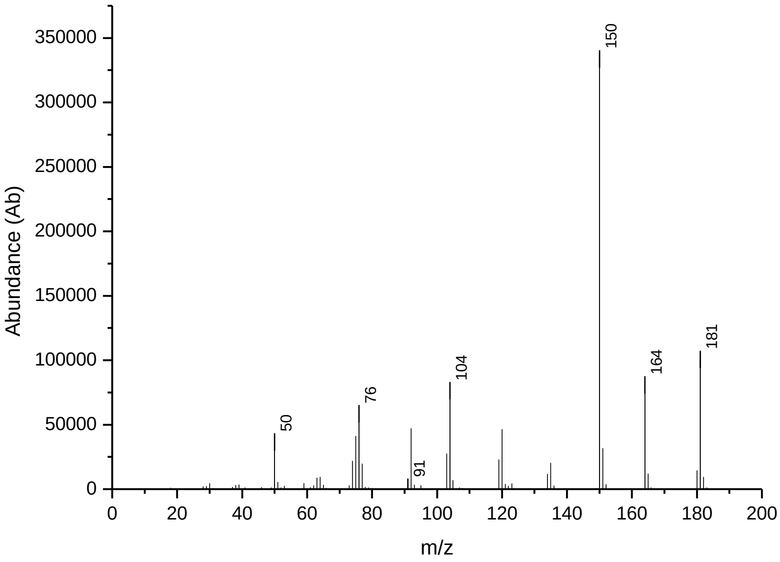

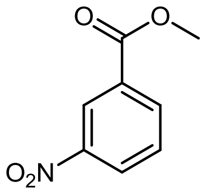

Methyl 3-nitrobenzoate (6)

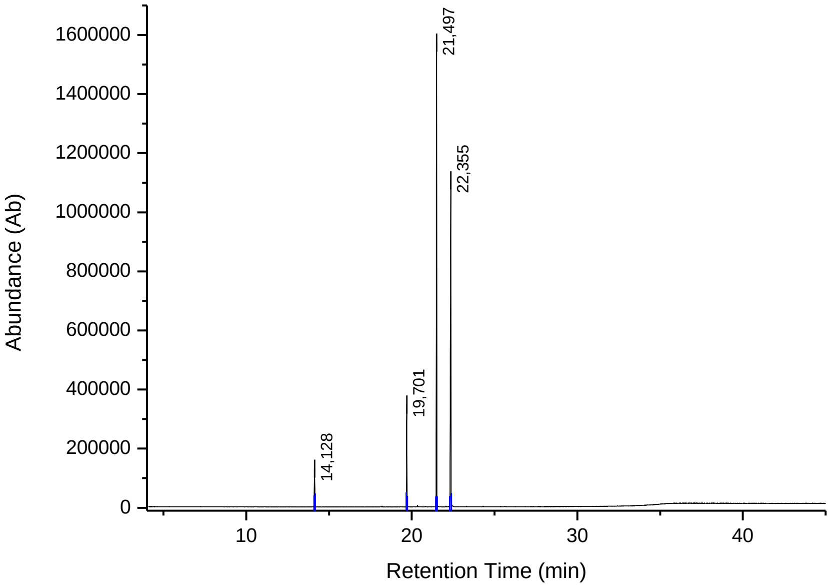

Peak	t_R	Area	% of Total
nitrobenzene	14.110	4064251	5
3-nitrobenzaldehyde	19.893	5098567	6
methyl 3-nitrobenzoate	21.751	49211596	60
3-nitrobenzylalcohol	22.206	23474229	29

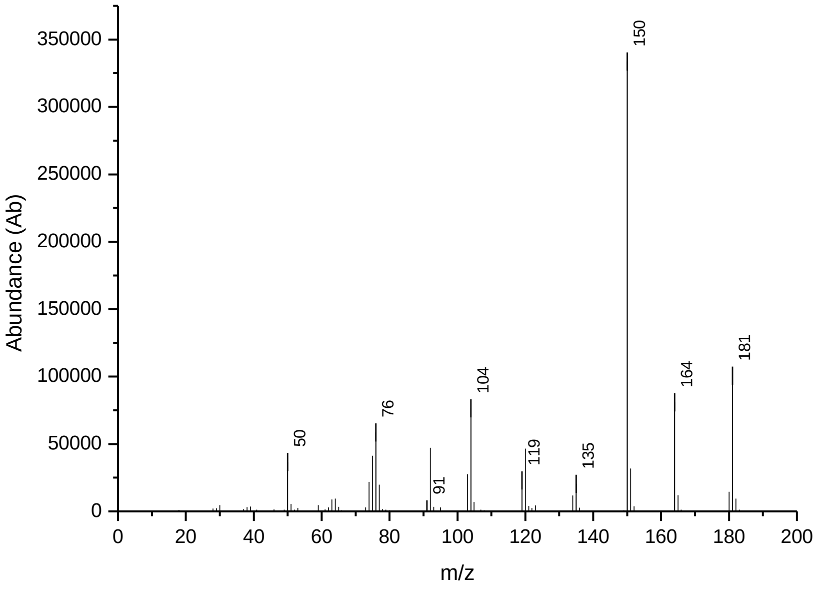

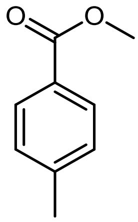

Methyl 4-methylbenzoate (7)

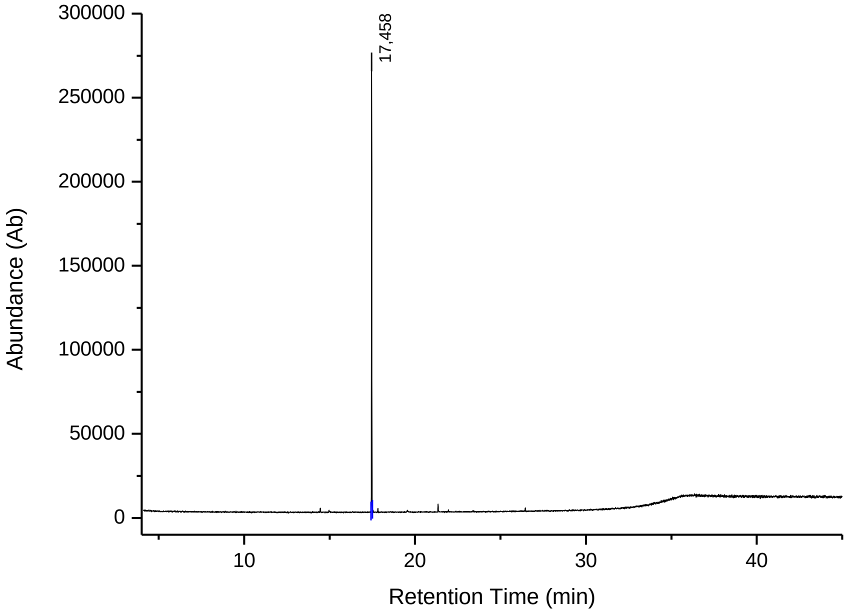

Peak	t_R	Area	% of Total
methyl 4-methylbenzoate	17.458	4331510	100

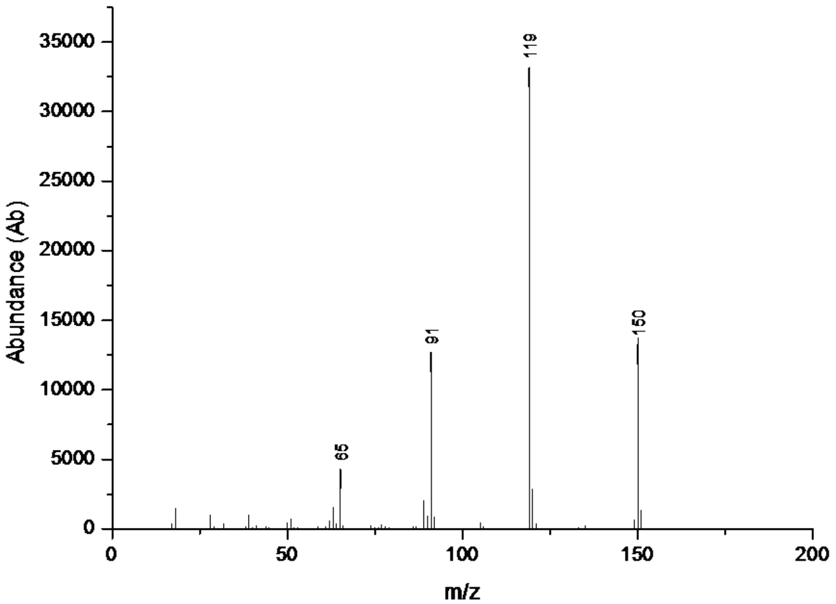

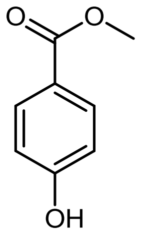

Methyl 4-hydroxybenzoate (8)

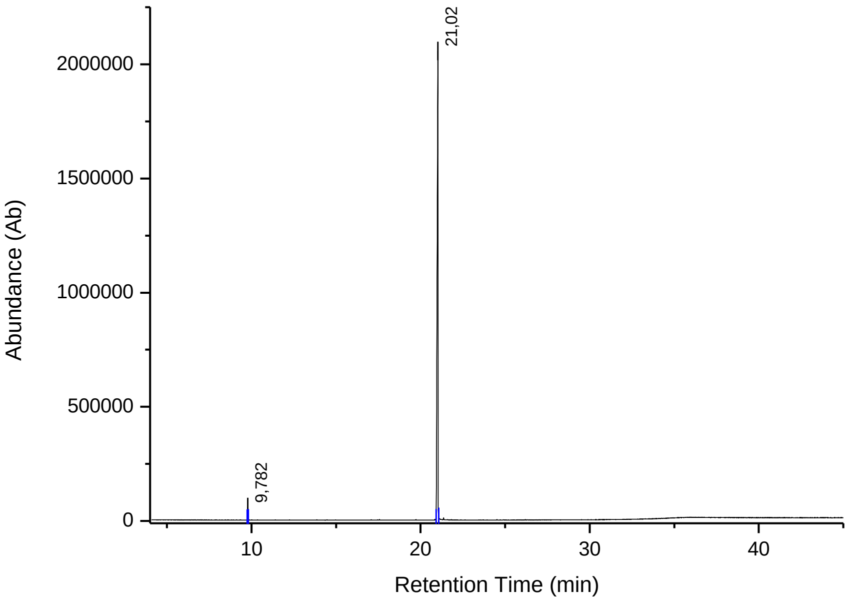

Peak	t_R	Area	% of Total
phenol	9.782	1559671	2
methyl 4-hydroxybenzoate	21.024	75112648	98

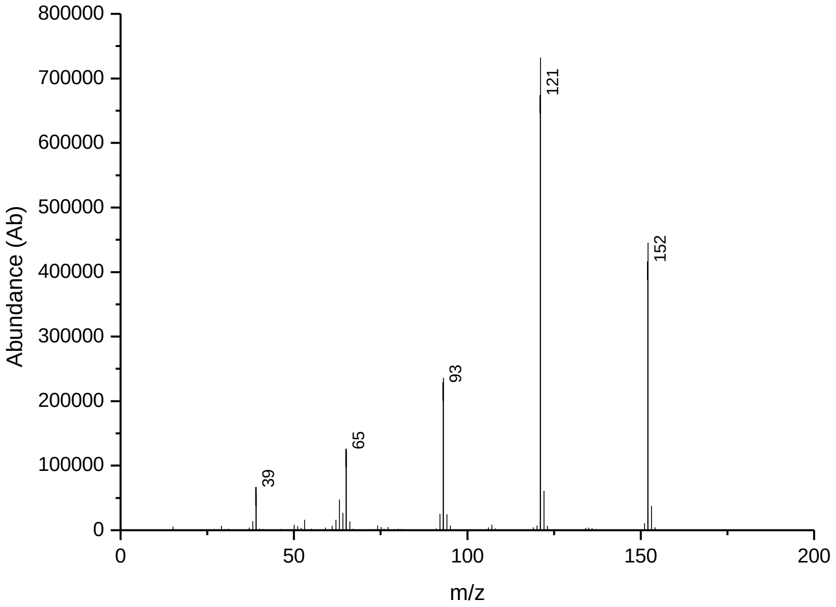

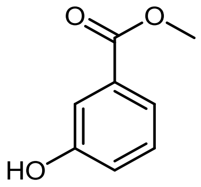

Methyl 3-hydroxybenzoate (9)

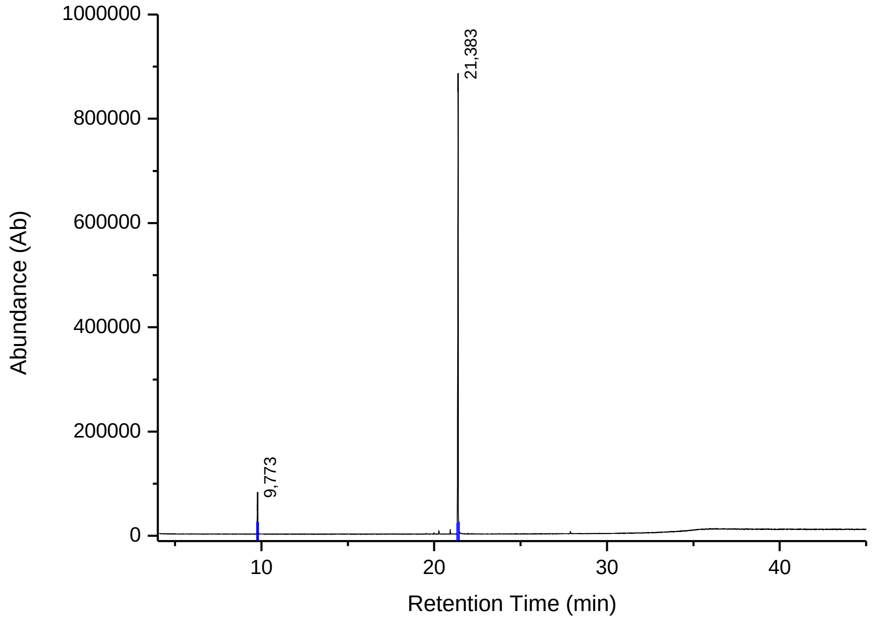

Peak	t_R	Area	% of Total
phenol	9.773	1671136	9
methyl 3-hydroxybenzoate	21.383	17862872	91

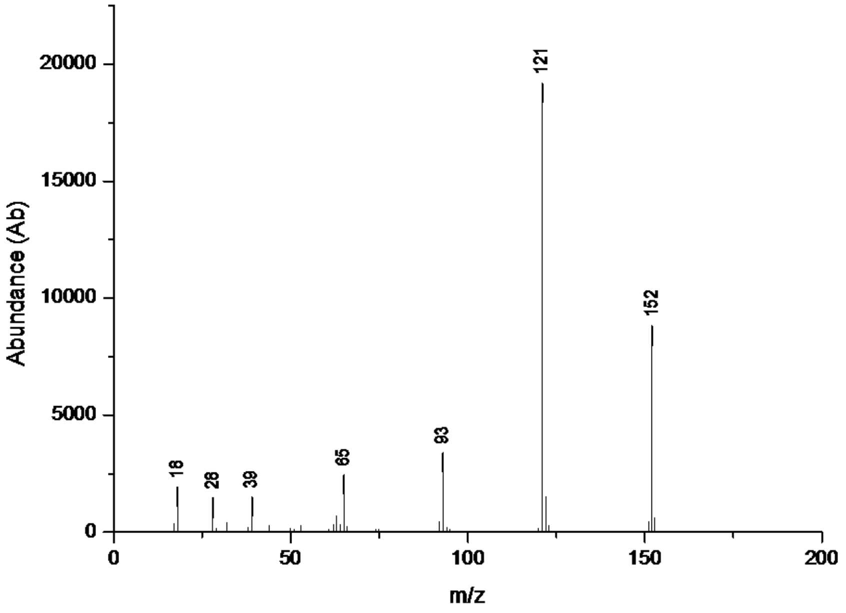

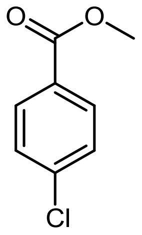

Methyl 4-chlorobenzoate (10)

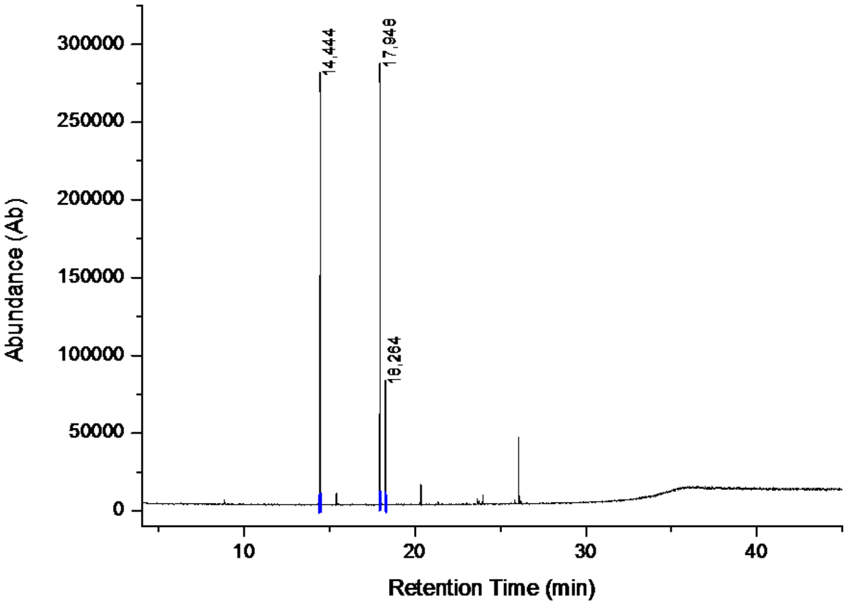

Peak	t_R	Area	% of Total
methyl benzoate	14.452	6239520	52
4-chlorobenzylalcohol	17.948	4638083	38
methyl 4-chlorobenzoate	18.264	1190777	10

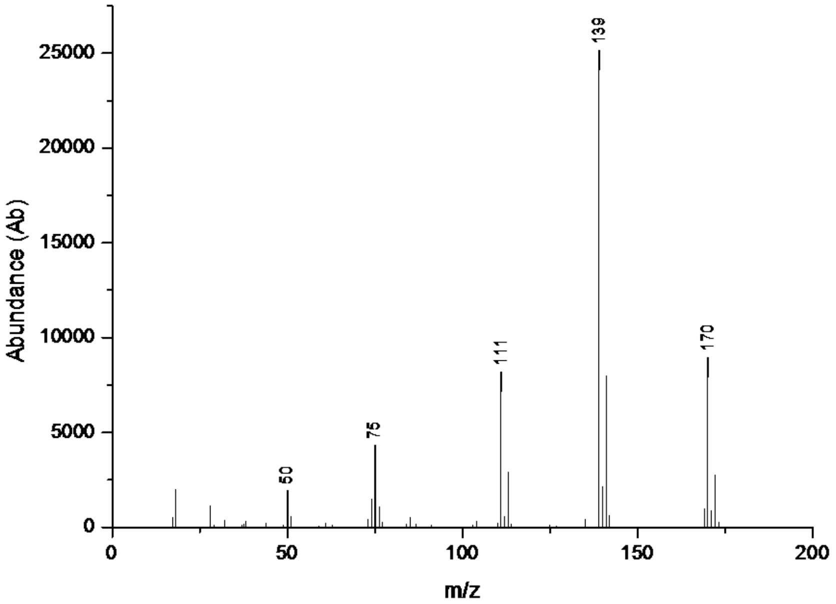

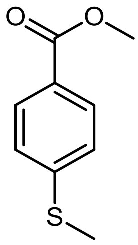

Methyl 4-(methylthio)benzoate (11)

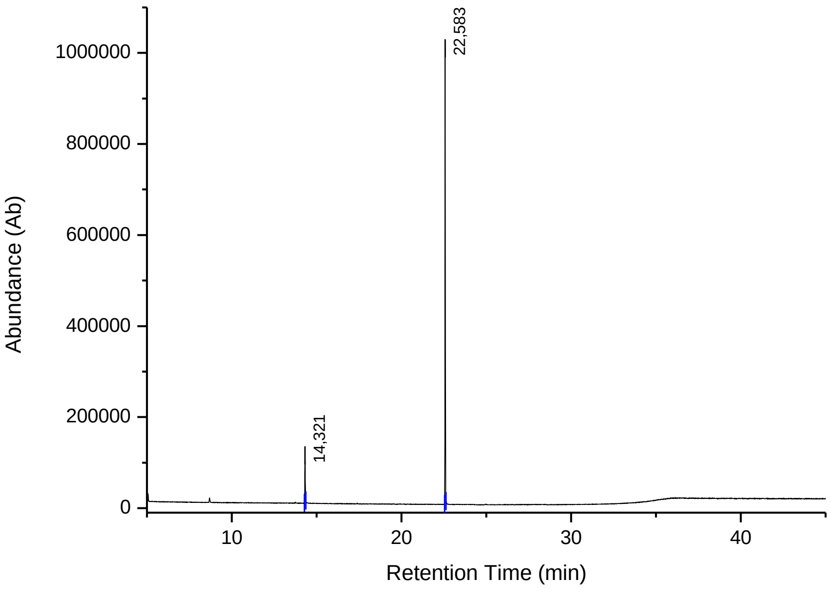

Peak	t_R	Area	% of Total
methyl benzoate	14.321	2561672	13
methyl 4-(methylthio)benzoate	22.583	16434629	87

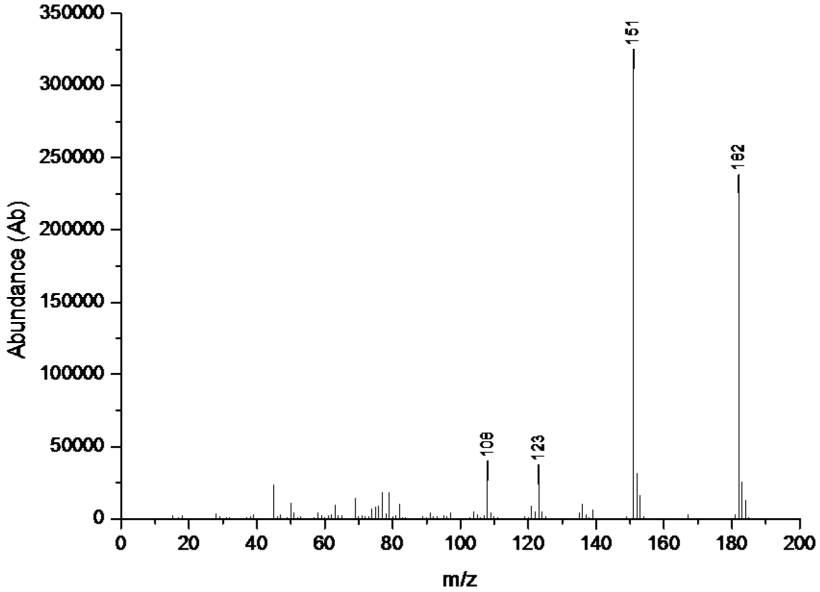

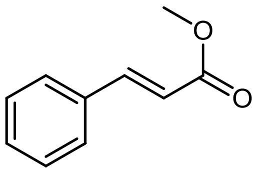

Methyl 3-phenylprop-2-enoate (12)

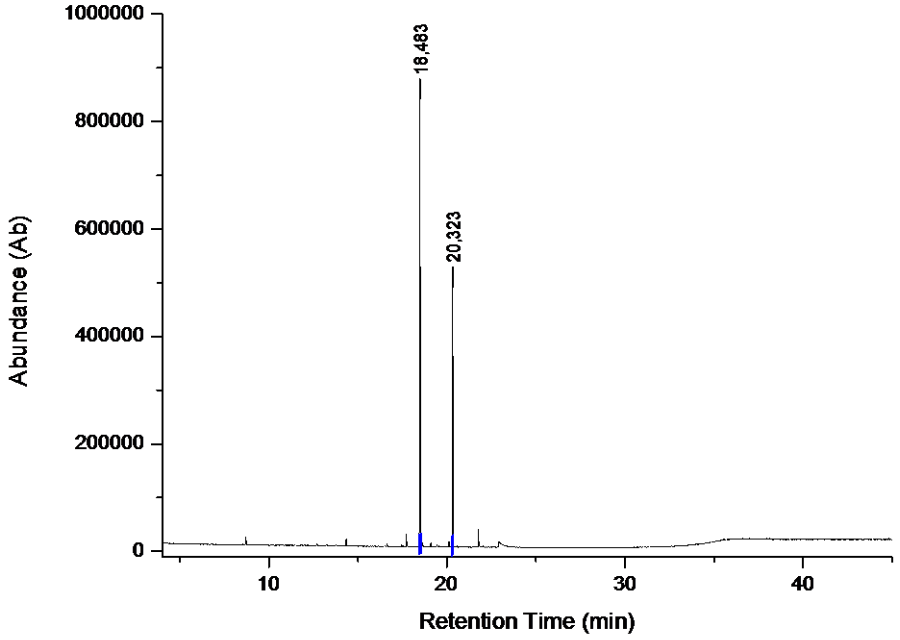

Peak	t_R	Area	% of Total
3-phenylprop-2-enol	18.492	14545844	63
methyl 3-phenylproprop-2-enoate	20.323	7586659	37

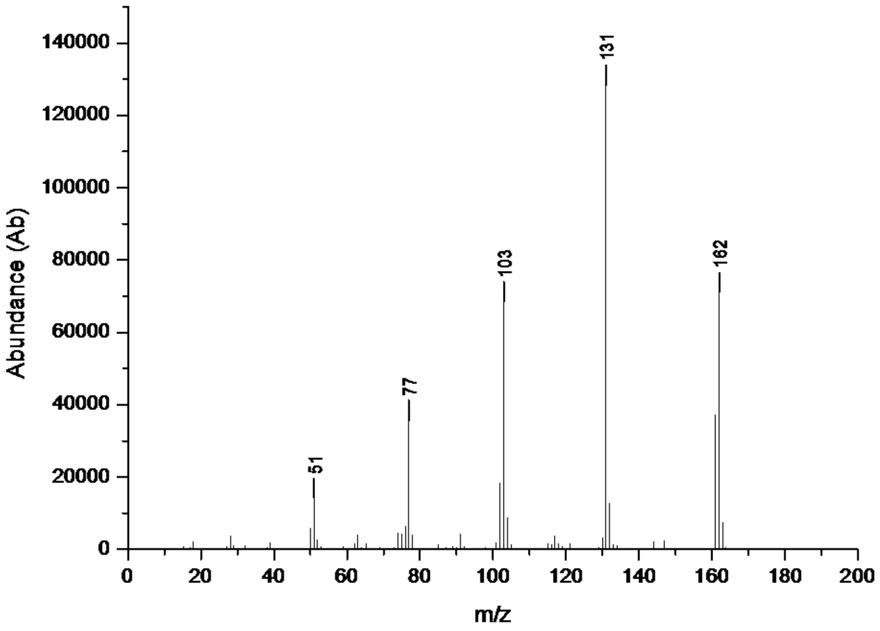

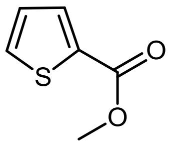

Methyl thiophene-2-carboxylate (13)

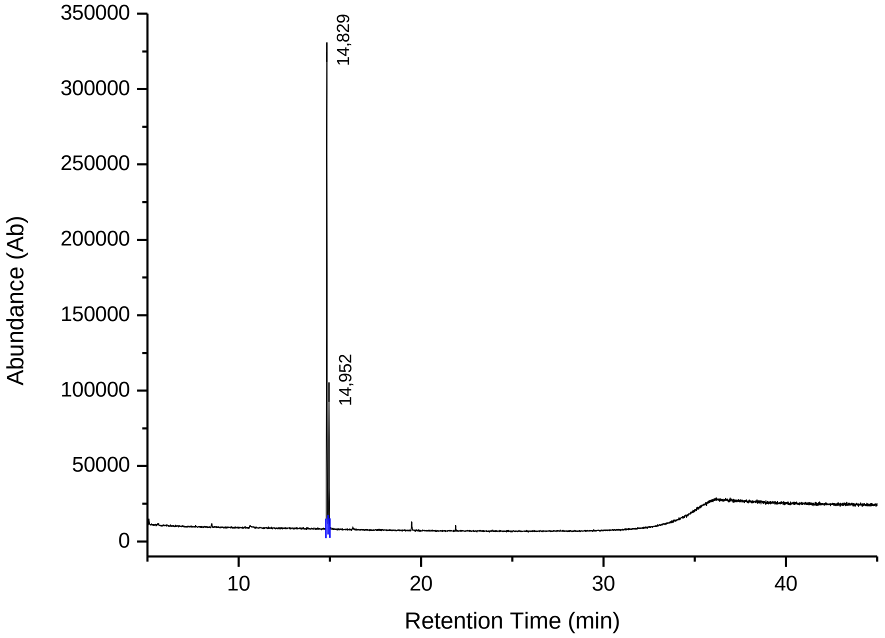

Peak	t_R	Area	% of Total
methyl thiophene-2-caboxylate	14.829	6922544	78
2-dimethoxymethyl thiopene	14.952	1977090	22

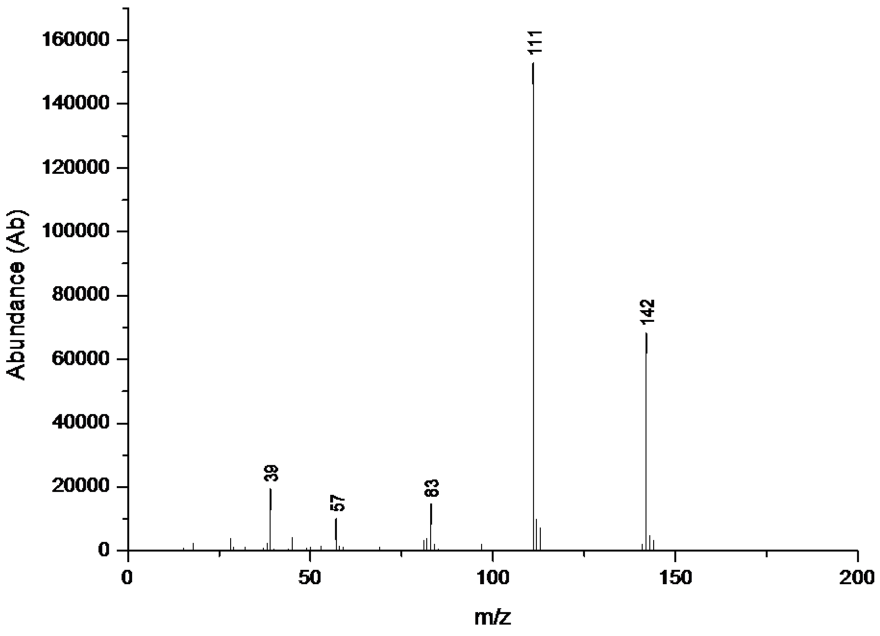

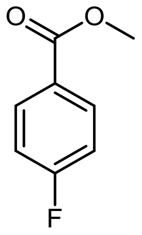

Methyl 4-fluorobenzoate (14)

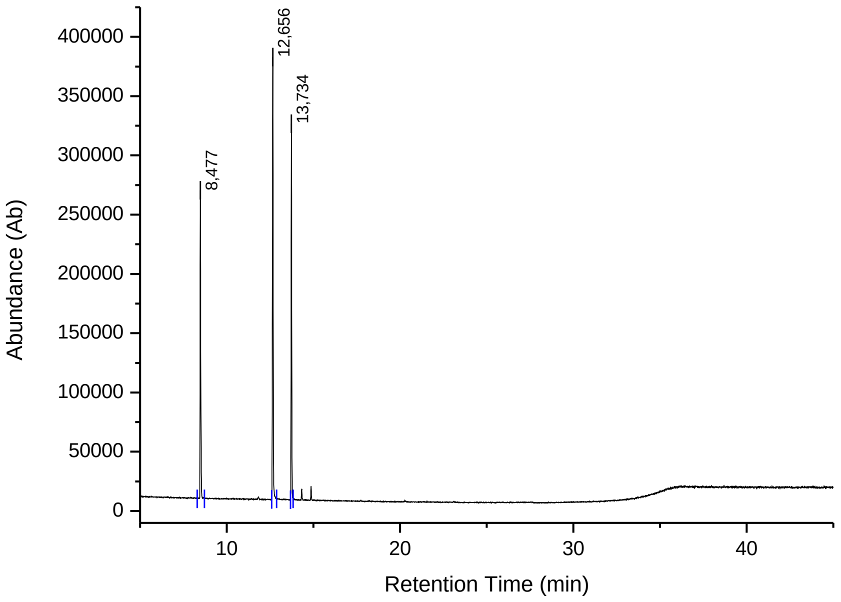

Peak	t_R	Area	% of Total
4-fluorobenzaldehyde	8.485	6786469	26
4-fluorobenzylalcohol	12.665	11355713	43
methyl 4-fluorobenzoate	13.734	8194213	31

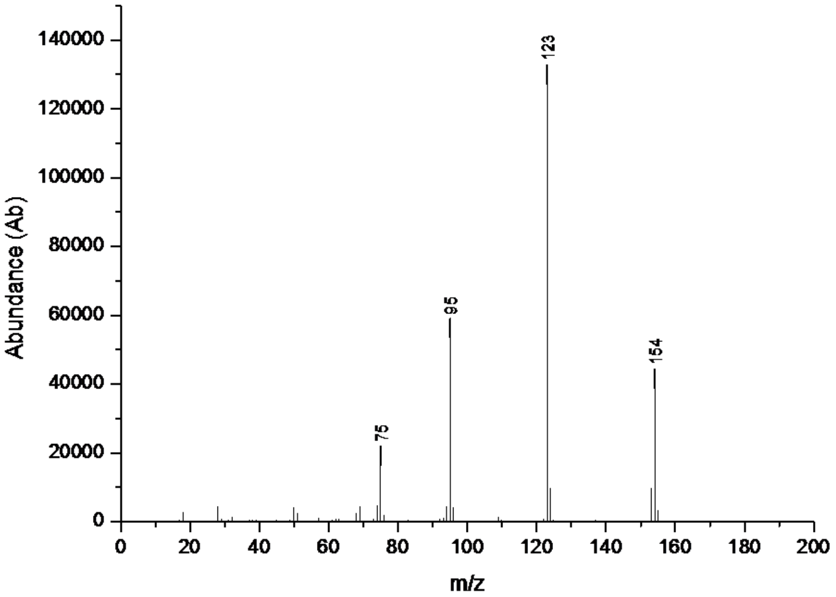

References

[1] A. B. Powell, S. S. Stahl, *Org. Lett.*, **2013**, *15* (19), 5072-5075.

[2] Spectral Database for Organic Compounds SDBS, SDBS #2319. Available at http://sdbs.db.aist.go.jp/sdbs/cgi-bin/direct_frame_top.cgi

[3] B. R. Kim, G. H. Sung, S.-G. Lee, *Tetrahedron*, **2013**, *69*, 3234-3237.

[4] C. Liu, J. Wang, L. Meng, Y. Deng, Y. Li, A. Lei, *Angew. Chem. Int. Ed.*, **2011**, *50*, 5144-5148.

[5] P. Li, J. Zhao, R. Lang, C. Xia, F. Li, *Tetrahedron Lett.* ,**2014**, *55*, 390-393.

[6] F. Mastronardi, B. Gutmann, C. O. Kappe, *Org. Lett.*, **2013**, *15* (21), 5590-5593.

[7] M. K. Agrawal, S. Adimurthy, P. K. Ghosh, *Synth. Commun.*, **2012**, *42*, 2931-2936.

[8] B. A. Tschaen, J. R. Schmink, G. A. Molander, *Org. Lett.*, **2013**, *15* (3), 500-503.
